# Neural and behavioural differences in multisensory statistical and reinforcement learning across development and task variants

**DOI:** 10.1162/IMAG.a.1117

**Published:** 2026-02-02

**Authors:** Nina Raduner, Carmen Providoli, Sarah V. Di Pietro, Maya Schneebeli, Marja A. Steuer, Sina Gubler, Ella Casimiro, Saurabh Bedi, Iliana I. Karipidis, Michael von Rhein, Nora M. Raschle, Christian C. Ruff, Silvia Brem

**Affiliations:** URPP Adaptive Brain Circuits in Development and Learning (AdaBD), University of Zurich, Zurich, Switzerland; Department of Child and Adolescent Psychiatry and Psychotherapy, University Hospital of Psychiatry, University of Zurich, Zurich, Switzerland; Jacobs Center for Productive Youth Development, University of Zurich, Zurich, Switzerland; Child Development Center, University Children’s Hospital Zurich, Zurich, Switzerland; Neuroscience Center Zurich, University and ETH Zurich, Zurich, Switzerland; Zurich Center for Neuroeconomics, Department of Economics, University of Zurich, Switzerland

**Keywords:** multisensory learning, surprise processing, reward prediction errors, computational modelling, fMRI, developmental cognitive neuroscience

## Abstract

The development and maturation of multisensory processing and integration depend on the brain’s ability to detect patterns and adjust behaviour based on feedback. However, the developmental dynamics of these mechanisms are not yet fully understood. This study examines neural and behavioural differences in multisensory statistical and reinforcement learning processes by comparing adults and children, and further investigates these processes across two learning tasks of varying difficulty in children. Using a discriminative choice and a match recognition multisensory associative learning task with embedded statistical stimulus regularities, 28 adults (19.0–30.9 years) and 2 groups of children (*N* = 28 and *N* = 29, 8.5–12.8 years) learned novel, non-linguistic associations of audio–visual or tactile–visual stimuli. Behaviourally, accuracy increased and reaction time decreased over time in all groups, and these learning effects were stronger in adults than in children and for audio–visual than for tactile–visual learning. Computational modelling revealed that adults employed more advanced learning strategies, with greater sensitivity to value differences, and more deterministic choice patterns. Behaviourally, reaction times were slower for more surprising trials across all groups. Neuroimaging showed that statistical surprise modulated activity in the lateral prefrontal cortex, intraparietal sulcus, and anterior cingulate cortex, while reward prediction errors were linked to striatal, medial frontal, and hippocampal regions. Reward prediction errors were consistent between adults and children and between task variants, whereas neural surprise processing emerged only in the match recognition task and showed developmental differences, indicating immaturity in children’s ability to detect statistical regularities. These findings highlight the importance of task design when comparing learning differences across developmental groups and showcase how reinforcement and statistical learning can be studied in parallel.

## 1 Introduction

Successfully navigating a multisensory environment requires the brain to integrate information from different modalities such as vision, audition, and touch (Stein et al., 2020; Van Atteveldt et al., 2014). Multisensory integration describes the process by which inputs from different senses are combined into a unified percept, enhancing perception, learning, and cognition (Barutchu et al., 2011, 2019; Birch & Belmont, 1965; Denervaud et al., 2020; Dionne-Dostie et al., 2015; Stein & Meredith, 1993; Stein & Stanford, 2008; Wallace et al., 2020). This integration plays a crucial role in language acquisition and other complex behaviours. Multisensory learning (MSL), in turn, refers to the formation of new associations between stimuli from different senses (Lauzon et al., 2022). It has been shown that successful MSL leads to the formation of new cross-modal associations, as shown through neuroimaging and behavioural studies (Butler et al., 2011; Lauzon et al., 2022). Understanding how MSL develops provides important insights into how the brain adapts to and learns from multisensory input (Shams & Seitz, 2008).

Learning can be broadly understood as the process by which organisms acquire the ability to predict future outcomes based on experiences (Niv & Schoenbaum, 2008). Two complementary frameworks describe this process: reinforcement learning (RL) and statistical learning (SL). RL refers to feedback-based learning, in which prediction errors drive adaptation of behaviour (Niv et al., 2012; Rescorla & Wagner, 1972; Sutton & Barto, 2005). In this type of learning, a prediction about the future success of an action is made and subsequently compared with the actual reward outcome; the discrepancy between prediction and outcome (known as reward prediction errors, RPEs) is used to update the reward expectation associated with a given action (Niv & Schoenbaum, 2008). This mechanism supports adaptive behaviour across the lifespan and is particularly important during childhood, when feedback plays a central role in learning contexts such as schooling. SL, by contrast, describes the ability to extract regularities and patterns from the environment without explicit feedback (Garrido et al., 2016; Ren & Wang, 2023; Saffran et al., 1996). Through SL, individuals can passively detect and learn relationships among stimuli based on their statistical structure (Finn et al., 2018; Garrido et al., 2016). This mechanism is especially relevant for early language acquisition, where children implicitly infer linguistic regularities from continuous sensory input (R. L. A. Frost & Monaghan, 2020; Romberg & Saffran, 2010). Although both RL and SL are driven by environmental information, they differ in the source of that information: RL depends on external reinforcement (Arbel et al., 2021) while SL relies on the detection of statistical contingencies in sensory input (R. Frost et al., 2019).

Computational modelling provides a powerful means to quantify these learning mechanisms and to link behavioural data with neural processes (for a review see Wilson & Collins, 2019). Specifically, the mathematical equations connect the observed data (decisions, reaction times, rewards) to the underlying learning models, by describing how hidden cognitive processes could have produced the measured data. Importantly, a fully generative model combines a learning process with a choice process, thereby integrating both learning and decision making within one framework (Mathys et al., 2011). In this study, we used two well-established computational models to capture these learning mechanisms: the Rescorla–Wagner (RW) model in combination with the drift diffusion model (DDM) for RL, and Shannon surprise for SL. In the RW model, learning occurs through trial-wise updating of expectations, with prediction errors weighted by a learning rate (Niv & Schoenbaum, 2008; Rescorla & Wagner, 1972; Subramanian et al., 2022). To model the choices depending on the expectations, we used the drift diffusion model (Ratcliff, 1978), which translates computational quantities such as expectations into behavioural and neural responses (Pedersen et al., 2017). By combining this model with the RW model, as proposed by Pedersen et al. (2017), we could thus uncover latent cognitive processes of multisensory reinforcement learning and the associated decision process and investigate how these are processed in the brain (Pedersen et al., 2017). Shannon surprise, in contrast, quantifies the unexpectedness of an event based on its probability of past observations (Konovalov & Krajbich, 2018; O’Reilly et al., 2013; Visalli et al., 2019). These models generate trial-wise computational signals that can be related to neural activity using fMRI.

Previous studies have demonstrated that RPEs reliably engage the ventral striatum, medial frontal cortex, and hippocampus (Cohen & Ranganath, 2005; Corlett et al., 2022; Fraga-González et al., 2025; Frei et al., 2025; Garrison et al., 2013; McClure et al., 2003; O’Doherty et al., 2003; Pagnoni et al., 2002) and negatively modulate activity in regions such as the anterior insula and cingulate cortex (Fouragnan et al., 2018; Raduner et al., 2025). Additionally, previous research has also demonstrated that reward prediction error processing generally does not differ between age groups (Hauser et al., 2015; Raduner et al., 2025; Van Den Bos et al., 2012). Studies investigating statistical learning linked more surprising events to slower reaction times (Kvålseth, 1987; Manning & Molesworth, 2023; Visalli et al., 2023). On a neural level, the Shannon surprise modulated the brain activation in the lateral prefrontal cortex (Konovalov & Krajbich, 2018; Visalli et al., 2019), the intraparietal sulcus (O’Reilly et al., 2013; Visalli et al., 2019), and the anterior cingulate cortex (Alexander & Brown, 2019; Hayden et al., 2011; Visalli et al., 2019), reflecting sensitivity to environmental unpredictability. Yet, while there is growing evidence in adults, the neural basis of these signals in children remains largely unknown.

To address this gap, we examined reinforcement and statistical learning during development using a multisensory associative learning paradigm during which participants learned novel, non-linguistic associations between either audio–visual or tactile–visual stimuli. We used two versions of this task, since during unpublished piloting for this study, we observed that younger children (<8 years old) struggled to learn effectively in one task version (match recognition task, commonly used in studies with adults). Thus, we introduced a second version (discriminative choice task) to investigate multisensory learning in children as young as 6 years old (Raduner et al., 2025), using a paradigm variant we have also successfully applied in earlier studies with children (Frei et al., 2025). In the present study, 2 age-matched groups of children (8.5–12.8 years) comprising 28 and 29 subjects completed 1 of the 2 tasks, while a group of 28 adults (aged 19.0–30.9 years) performed the match recognition task. By including both tasks and comparing age-matched groups, we aimed to directly assess how task design influences multisensory processing and learning, both behaviourally and neurobiologically. Including an adult group in the study design enables us to disentangle developmental effects from task-related effects, thereby providing a clearer understanding of multisensory learning across development. In this context, we define reward prediction errors (RPEs) as the core computational signal underlying reinforcement learning, reflecting the difference between expected and received outcomes. In contrast, statistical learning quantifies the unexpectedness of a stimulus based on its probability of occurrence and is measured by the Shannon surprise (an information-theoretic measure; surprise). Although statistical learning has been examined in children, only one study to date has investigated Shannon surprise in this age group, linking it to pupillary responses (Colantonio et al., 2023). Here, we extend this work by examining the neural correlates of Shannon surprise in children and adults during multisensory associative learning. Previous adult studies have typically investigated surprise and RPE within complex sequential decision-making tasks (e.g., Liakoni et al., 2022; Möhring & Gläscher, 2023). In contrast, our study applies these learning signals to a simpler associative learning paradigm with a single decision stage (based on the approach established by Bedi et al. (2025)), making it well suited for developmental populations.

This study had two main objectives: First, we aimed to investigate **differences in statistical and reinforcement learning between adults and children, at the level of behaviour and neural processes**. Building upon these findings, our second aim was to **compare statistical and reinforcement learning in children across two different task versions**. Behaviourally, we expected that adults would generally outperform children, consistent with previous developmental studies of associative learning (Master et al., 2020). In our unpublished pilot data, we observed higher performance in the discriminative choice task than in the match recognition task in younger children (<8 years old). In this study, we investigate whether this task-specific performance difference also occurs in older children (>8 years old), hypothesizing better performance in adults than in children and potentially between task versions in children. Further, we expected slower reaction times for more surprising trials (Bedi et al., 2025; Kvålseth, 1987; Manning & Molesworth, 2023; Visalli et al., 2023), in both adults and children and both task versions, reflecting increased processing demands. Additionally, we explored potential differences in the modelling parameters related to learning between adult and child groups and task versions, which could reveal distinct strategies or processing mechanisms. Finally, at the neural level, we predicted that RPEs would positively modulate activity in striatal, medial frontal, and hippocampal regions (Corlett et al., 2022; Fraga-González et al., 2025; Raduner et al., 2025), and negatively modulate the anterior insula and cingulate cortex (Fouragnan et al., 2018; Raduner et al., 2025). We anticipated no significant differences in the neural RPE representation between adults and children (Hauser et al., 2015; Van Den Bos et al., 2012) or between task versions (Corlett et al., 2022). In contrast, surprise was expected to engage the lateral prefrontal cortex, the intraparietal sulcus, and the anterior cingulate cortex in adults (Alexander & Brown, 2019; Hayden et al., 2011; Konovalov & Krajbich, 2016; O’Reilly et al., 2013; Visalli et al., 2019) with potential differences in children and between task versions.

Thus, by linking computational models with behavioural and neuroimaging data, our study aims to provide a comprehensive account of how statistical and reinforcement learning mechanisms develop and interact during multisensory learning.

## 2 Material and Methods

### 2.1 Participants

For this study, 36 adults (27 female, 9 male) completed the MSL task, but 8 females were randomly excluded using the slice_sample-function from the dplyr package. This was done first to match the group size of the children’s groups and second to reduce the imbalance between females and males in the adult group. The final adult sample included 28 healthy adults (*M* = 25.13 ± 2.98 years, 19 female). Twenty-four participants of the adults were right handed, one ambidextrous, and the remaining three participants were left handed. The adult participants were recruited in a Swiss University setting, of which 20 were either currently students or had already obtained a bachelor’s, master’s, or doctoral degree. From the 29 children who completed the match recognition task, we included 28 in the first group of children (1 subject had to be excluded from the analyses due to too much movement (20.9% corrupted scans) during the AV task (see Section 2.3)). Additionally, from all the children who completed the discriminative choice task (*N* = 67, see Supplementary Material and Raduner et al. (2025)), an age-matched subsample was selected as the second group of children (*N* = 29). Note that all but two children included in the group completing the discriminative choice task were also part of the study sample reported by Raduner et al. (2025). In total, we included 57 healthy children (*M* = 10.22 ± 1.19 years, 26 female) in this study. All children had mean IQ scores exceeding 80 (see Table 1). All participants were either German or Swiss German speaking and had normal or corrected-to-normal vision. The Edinburgh Handedness Scale (EHI; Oldfield, 1971) was used to assess the handedness of each participant. See Table 1 for the demographics in each group. Baseline cognitive characteristics did not differ between the two groups of children (see Supplementary Table S1 for details).

**Table 1.** Demographic characteristics of study participants.

	Match recognition		Discriminative choice	
	Adults	Children	Children	*p*
*N*	28	28	29	
Age [y] *M* (*SD*)	25.13 (2.98)	10.26 (1.25)	10.22 (1.16)	<.001
Age range [y]	19.0–30.9	8.5–12.8	8.9–13	
Gender [f:m]	19:9	8:20	17:12	.009**
Handedness^a^ [l:a:r]	3:1:24	3:0:24	1:1:27	.727
Mean IQ^b^ *M* (*SD*)	111.6 (7.6)	108.2 (12.6)	105.6 (10.2)	.191

*Note*. **Age:** Kruskal–Wallis test, followed by Dunn’s post hoc tests (significant difference between the adult group and the two children groups, no difference between the children groups). **Gender:** We used Fisher’s Exact Test to account for differences between the groups and gender distribution did significantly differ across groups, followed by Bonferroni corrected post hoc tests (significant difference between the adult group and the children group completing the match recognition task [*p* = .021]; a trend for a difference between the two groups of children [*p* = .099]). Thus, gender was added as a covariate in all analyses (see Section 2.5). **Handedness:** We used Fisher’s Exact Test to account for the differences in the handedness distribution between the groups, which did not differ. **IQ:** The mean IQ value was estimated using each participant’s verbal and non-verbal IQ estimation. The verbal IQ was estimated by the Peabody Picture Vocabulary Test—fourth edition (PPVT-IV; Lenhard et al., 2015) and the non-verbal IQ using the matrix reasoning and the figure weights subtests from the Wechsler Intelligence Scale for Children—fifth edition (WISC-V; Wechsler, 2017) or the Wechsler Adult Intelligence Scale—fourth edition (WAIS-IV; Petermann, 2012; Wechsler, 1970). We performed a Kruskal–Wallis test to compare mean IQ values between the groups, which showed no significant differences. **p ≤ .01.

a Data missing from one child.

b IQ data missing for nine adult participants.

Parents or guardians of all but six children filled out an in-house questionnaire on the child’s language development and the Child Behavior Checklist (CBCL/6-18R, Döpfner et al., 2014). One child had a diagnosis of attention deficit hyperactivity disorder (ADHD). Additionally, two children had a diagnosis of developmental language impairments (developmental language delay or dyslexia). We observed clinically significant T-values (>63) as a total score on the CBCL/6-18R in nine children, including the one with a diagnosis of ADHD. The mean CBCL/6-18R scores did not differ between the two child groups (*M*_matchRecognition_ = 52.44, *M*_discriminativeChoice_ = 53.89, *t*(49.23) = -0.54, *p* = .590). Of note, the CBCL/6-18R was completed as a parent- or guardian-reported measure and was not verified by a clinician; therefore, the results may be subject to reporting bias. In total, 11 children across both groups of children had either a formal clinical diagnosis, scores above the threshold on the CBCL questionnaire, or both. Oral assent was obtained from all children, and written consent was provided by a parent or guardian and the adult participants. The study received approval from the local ethics committee of the Canton of Zurich and neighbouring cantons in Switzerland (BASEC-Nr: 2022-01368). Participants were compensated with vouchers and gifts as a token of appreciation for their involvement.

### 2.2 Experimental design

In this study, each participant completed an audio–visual and tactile–visual multisensory learning task embedded in a child-friendly story. The tasks were based on the approach introduced by Bedi et al. (2025) and used stimuli established by Frei et al. (2025) and Fraga-González et al. (2025). Participants learned multisensory associations through trial-by-trial feedback, pairing visual symbols displayed on rocket images with either auditory or tactile stimuli. These visual symbols served as unique identifiers for individual rockets. During trials, a sound or vibration was presented simultaneously with either a single rocket including a symbol (match recognition task) or two rockets with distinct symbols (discriminative choice task). Children were instructed that sounds and vibrations correspond to specific rocket engines. In the discriminative choice task, participants selected the rocket that matched the given sound or vibration by pressing the corresponding button. In the match recognition task, they indicated whether the presented stimulus pair was matching or non-matching by pressing the corresponding button. Responses were followed by immediate feedback, which participants were instructed to use to learn the associations over time. Participants were told to collect as many stars as possible (rewarded during positive feedback) and thus incentive feedback was included in our tasks. Although the feedback structure was probabilistic, this was only implicitly communicated to participants (e.g., “sometimes a different engine might be in the rocket making a different sound than usual”).

The discriminative choice task was the primary version used with children, particularly as younger participants (<8 years old) encountered difficulties with the match recognition task. However, while the discriminative choice task was better suited for younger children, data from older children revealed ceiling effects, limiting its utility for capturing individual differences or developmental changes in this subgroup. To address this limitation, and to ensure an appropriate level of task difficulty across the full age range, an alternative match recognition task was introduced. Comparing performance across these two task versions allows for the examination of how task structure influences learning processes and neural responses and helps to disentangle age-related changes from task-specific effects. This approach further makes it possible to validate findings across different task demands, ensuring the robustness and generalizability of the observed developmental trajectories in multisensory learning. Both task versions were used in the final analyses. The discriminative choice task was developed based on unpublished pilot observations but was fully implemented in the final study alongside the match recognition task, to allow for comparisons across age and task design.

Each child completed the audio–visual and tactile–visual multisensory learning task outside of the MRI scanner during a behavioural testing session on average 10.53 days (*SD* = 8.40 days) before the MR session. During the neuroimaging session, the children repeated the tasks with different (unknown) symbols, sounds, and vibration patterns in the MRI scanner. The adults, however, only completed one audio–visual behavioural run at the beginning of the neuroimaging session. The behavioural runs were presented using PsychoPy® software (Version 2022.1.1, Peirce et al. (2019); https://www.psychopy.org/) on a notebook connected to a tactile stimulation device (mini-PTS piezoelectric stimulator device from dancer design, http://www.dancerdesign.co.uk/), stimulating the right pinkie. For the MR runs, we used MR-compatible over-ear headphones (MR confon GmbH, Magdeburg, Germany, http://www.mr-confon.de/de/) and the task was presented on goggles (CinemaVision 20/20, Resonance Technology, Northridge, CA). During the MR session, the sound volume and goggles settings were adjusted individually to make sure that each participant could perceive the stimuli well.

The experimental protocol required each participant to complete one run of each modality in the MR scanner, but since some of the participants were children, not all participants could complete all tasks according to the experimental protocol. Factors such as excessive movement, lack of motivation (too many omissions, see Section 2.3), or fatigue could lead to incomplete or unusable data. In such instances, we aimed to repeat the runs with insufficient data quality using new stimulus sets, whenever possible. For this study, we included data of the first audio–visual and the first tactile–visual MR-run for each participant with sufficient data quality, resulting in two MR-runs per subject.

#### 2.2.1 Discriminative choice task

**Fig. 1.**
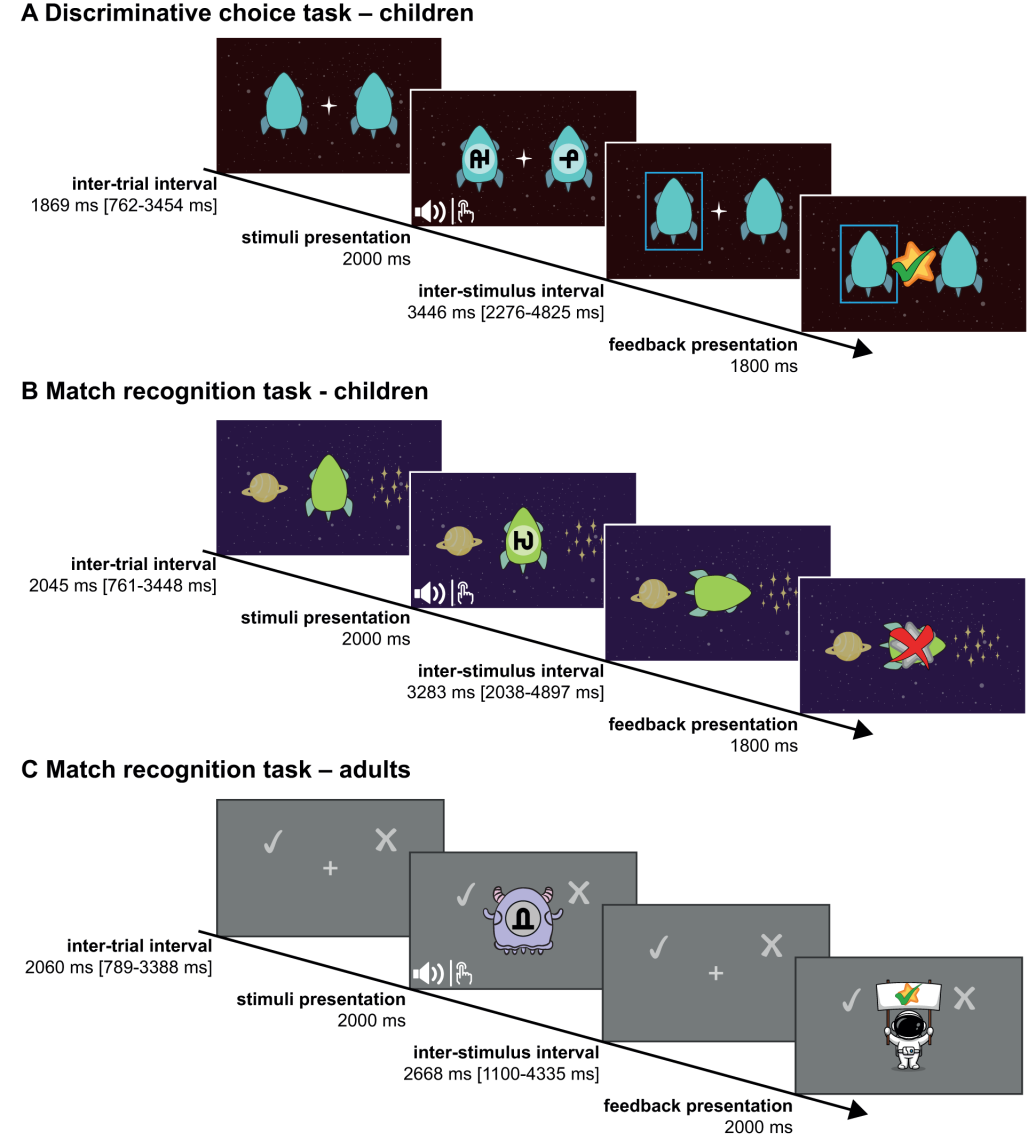
Overview of the different task versions used in this study. (A) The task structure of the discriminative choice task used in children. (B) The match recognition task used in children and (C) the version used in adults.

The discriminative choice task has been previously described in Raduner et al. (2025). Please note that the data of all but two children here were also part of the group analysed in an earlier manuscript (Raduner et al., 2025). Subsequently, the main differences between the two task versions are summarized. In the discriminative choice task, two symbols were presented simultaneously with either a sound or vibration. The participant then had to indicate whether the sound/vibration corresponded to the rocket presented on the left- or right-hand side, each identifiable with a distinct symbol, by pressing the corresponding button (see Fig. 1A). The mean duration of the inter-trial interval (ITI) was 1.87 seconds [0.76–3.45 seconds] and 3.45 seconds [2.28–4.83 seconds] for the inter-stimulus interval (ISI). The ITI and ISI both followed a gamma distribution. The timing of the ISI and ITI was changed after the first six participants to prolong decision time. For these participants, the ITI had a mean duration of 2.06 seconds [0.79–3.39 seconds] and the ISI of 2.67 seconds [1.11–4.34 seconds]. The ITI and ISI were optimized using task design efficiency analyses to decorrelate stimulus and feedback processing (no correlation between stimulus and feedback regressors exceeded 30%). One run consisted of 44 trials, resulting in a total task duration of 401 seconds or 384 seconds for the initial six participants.

During each run, participants were presented with 4 visual and 4 auditory or tactile stimuli, resulting in 16 possible combinations, 4 of which were matching. The stimuli used were the same as those used and described in Raduner et al. (2025). The visual symbols were chosen from the PseudoSloan font (Vildavski et al., 2021) and grouped into sets based on their complexity. Each set consisted of one low complex, two medium complex, and one high complex stimulus (see Raduner et al. (2025) for a more detailed description). Auditory stimuli were drawn from environmental sounds available in the Mixkit sound effects database (https://mixkit.co/free-sound-effects/). We selected sounds that were difficult to identify or name. Each sound was trimmed to a duration of 2 seconds and normalized using Audacity® recording and editing software (version 3.5.1; Team Audacity, 2024). The sounds were then grouped into sets based on the spectral entropy of each sound (see Raduner et al. (2025) for a more detailed description), such that each set consisted of one sound with low, two with medium, and one with high complexity. Lastly, we developed three distinct sets of tactile stimuli. Each set included four unique vibration patterns, each lasting 2 seconds. Two patterns in each set had stable, distinct frequencies (low complexity), one followed an increasing or decreasing frequency pattern (medium complexity), and one had an (inverted) U-shaped frequency pattern (high complexity; see Appendix A1). The manipulation of the complexity of stimuli within the set was done to enhance the distinguishability of the individual stimuli.

To be able to investigate surprise and statistical learning, the presentation frequency of stimulus combinations was adjusted within runs so that certain triplet (two symbols and a sound/or vibration) combinations occurred more frequently than others. This approach was based on Bedi et al. (2025). Each visual, auditory, or tactile stimulus was presented an equal number of times (22 out of 44 trials for visual stimuli, and 11 out of 44 for auditory/tactile stimuli), but the pairing between modalities varied. The distribution of these pairings is detailed in Table 2. The combinations paired at 100% indicate the matching pairs (A0, B1, C2, D3), meaning that when auditory/tactile stimulus A was presented, visual stimulus 0 was always paired with it. However, in 63.3% (7 out of 11) of trials involving stimulus A, visual stimulus 1 appeared as the second visual stimulus, with visual stimulus 2 appearing in 27.3% (3 out of 11) of trials, and visual stimulus 3 in 9.1% (1 out of 11) of those trials.

**Table 2.** Presentation frequency of the multisensory stimulus combinations in the discriminative choice task as manipulation of the surprise.

	Visual			
Auditory/tactile	0	1	2	3
A	100%	63.6%	27.3%	9.1%
B	63.6%	100%	9.1%	27.3%
C	27.3%	9.1%	100%	63.6%
D	9.1%	27.3%	63.6%	100%

*Note.* This table shows the presentation frequencies of the multisensory stimulus combinations in the discriminative choice task. Auditory/tactile stimulus A was consistently paired with visual stimulus 0. In 63.6% of the trials involving auditory/tactile stimulus A, visual stimulus 1 appeared as the second (non-matching) visual stimulus, while visual stimulus 2 appeared in 27.3% and stimulus 3 in 9.1% of the trials as the non-matching visual stimulus. With this manipulation, some multisensory stimulus triplets appeared more often than others and, therefore, the statistical properties of the environment were manipulated as a basis for the surprise signal.

#### 2.2.2 Match recognition task

We aimed to keep the match recognition task as similar as possible to the discriminative choice task. They differed in the following aspects: instead of two rockets with one symbol each appearing together, only one rocket with one symbol was displayed in the middle of the screen during stimulus presentation. Left and right of the rocket, a planet and stars were displayed, signifying the matching side (planet) and non-matching side (stars; see Fig. 1B). Children were told that the rocket could only return home to its planet (matching pairs) when the correct sound or vibration was presented along with it; otherwise, the rocket would remain in space (non-matching pairs). The participants then had to indicate whether the presented audio–visual or tactile–visual pair was matching by pressing the button on the corresponding side. Stimulus and feedback duration did not differ between the tasks. In this version, the ITI and ISI were also gamma distributed with a mean duration of 2.05 seconds [0.76–3.45 seconds] and 3.28 seconds [2.04–4.90 seconds], respectively. The ITI and ISI were optimized using task design efficiency analyses to decorrelate stimulus and feedback processing (no correlation between stimulus and feedback regressors exceeded 30%). The match recognition task consisted of 42 trials per run, resulting in a total task duration of 383 seconds. This task version only consisted of three stimuli per modality and run, resulting in nine possible combinations of which three were matching. We created the same number of stimuli sets and applied the same rules when creating the sets as for the discriminative choice task. However, for the visual and auditory sets, we only included one stimulus with medium complexity per set, and the tactile sets only included one stable frequency instead of two.

Although the basic task design was the same for the adults and the children, the presentation varied slightly, as depicted in Figure 1C. The visual symbols were presented on the backside of the aliens next to a tick and cross on the background, indicating the matching and non-matching sides, respectively. Here, the participants were told that the different aliens make different sounds and dances (the vibrations they felt on their finger) and that they had to indicate whether the sound/vibration came from the presented alien (matching) or a different alien that is not presented (non-matching). The goal was to learn which sound and vibration belonged to which alien. In this version, feedback was presented for 2 seconds instead of 1.8 seconds. The ITI and ISI were also gamma distributed: 2.06 seconds [0.79–3.39 seconds] and 2.67 [1.11–4.34 seconds], respectively. The ITI and ISI were optimized using task design efficiency analyses to decorrelate stimulus and feedback processing (no correlation between stimulus and feedback regressors exceeded 30%). This version also included 42 trials and resulted in a task duration of 367 seconds.

We also manipulated the presentation frequency of the stimulus pairs to investigate statistical learning in this task, in line with the approach established by Bedi et al. (2025). Here too, each visual, auditory, and tactile stimulus appeared equally often (14/42 trials per stimulus) and in 50% of the trials, matching pairs were presented. But some of the non-matching pairs appeared more often (15/42 trials, 35.7%) than others (6/42 trials, 14.3%). For example, auditory stimulus A appeared seven times with the visual stimulus 0 (matching combination) and five times with visual stimulus 2, but only two times with visual stimulus 1. All the distributions of pairings are given in Table 3.

**Table 3.** Presentation frequency of the multisensory stimulus combinations in the match recognition task as manipulation of the surprise.

	Visual			
Auditory/tactile	0	1	2
A	50%	14.3%	35.7%
B	35.7%	50%	14.3%
C	14.3%	35.7%	50%

*Note.* This table gives the presentation frequencies of the multisensory stimulus combinations in the match recognition task in both adults and children. Auditory/tactile stimulus A was paired with visual stimulus 0 in 50% of trials. In 35.7% of the trials, the auditory/tactile stimulus A was paired with the visual stimulus 2. In the remaining 14.3% of trials, it was paired with the visual stimulus 1. With this manipulation, some multisensory stimulus pairs appeared more often than others and, therefore, the statistical properties of the environment were manipulated as a basis for the surprise signal.

#### 2.2.3 Manipulation of task difficulty

The task performances from the behavioural runs were used to calibrate the task difficulty in the MR scanner, ensuring it remained appropriately challenging. Adjustments were made by altering the trial structure or incorporating probabilistic feedback. In most trials, participants received correct feedback (positive after correct and negative after incorrect responses). However, in a predefined proportion of trials (5%, 10%, or 20% per run, depending on pre-run performance), the feedback valence was intentionally reversed (incorrect feedback), such that correct choices were followed by negative feedback and incorrect choices by positive feedback. These trials with incorrect feedback were evenly distributed across the auditory and tactile stimuli within each run to maintain balanced task difficulty. Neutral feedback was consistently provided for omitted responses. Additional details regarding trial structure, adaptive rules, and feedback implementation are described in Appendix A2.

The following variables were pseudorandomized for all participants: the order of tasks (AV and TV), the trial structures (order of stimulus presentation), the specific visual, auditory, and tactile sets to be used, and the assignment of stimuli for assembling the four correct combinations in each run. Further details regarding the trial design and probabilistic feedback are given in Appendix A2.

### 2.3 MRI data acquisition and preprocessing

The MRI data were recorded using a 32-channel coil on a Philips Achieva 3 Tesla scanner (Best, The Netherlands). A whole-brain gradient echoplanar image (EPI) sequence was used to acquire T2*-weighted functional images. The EPI sequence included five dummy scans and was then followed by 291 dynamic scans for the discriminative choice task, 278 for the match recognition task in children, and 268 for adults. For both task versions, the acquisition parameters were as follows: repetition time (TR) = 1.395 seconds, echo time (TE) = 35 ms, with 44 slices, a voxel size of 3.0 × 3.0 × 3.0 mm³, and a slice gap of 0.3 mm. The matrix size was 64 × 62 pixels, with a flip angle of 80°, a multiband factor of 2, a sofTone factor of 2, and a SENSE reduction factor of 2. As a result of timing modifications in the discriminative choice task (see Section 2.2), the EPI sequence for 6 participants completing the discriminative choice task included 278 dynamic scans, with all other parameters remaining identical. A magnetization-prepared rapid acquisition gradient echo (MPRAGE) sequence was used to acquire T1-weighted anatomical images, which were used for co-registration. The acquisition parameters were as follows: TR = 6.96 ms, TE = 3.2 ms, aligned at AC-PC, flip angle = 9°, voxel size = 1.0 × 1.0 × 1.0 mm³, field of view = 270 × 255 mm², number of slices = 176. Preprocessing, including slice-time correction, realignment, co-registration, segmentation, and whole-brain analyses, was done with MATLAB (version R2020b, MathWorks Inc, 2020) toolbox SPM12 (7219, W. Penny et al., 2007). A paediatric tissue probability map (TPM), which was matched for age and gender, was created using the Template-O-Matic toolbox (Wilke et al., 2008) and used for tissue segmentation for the two children’s groups. Using custom-made TPMs for children has proven to improve segmentation in children (Wilke et al., 2008). For the adults, the SPM’s default TPM was used. All data were then normalized to the MNI space. Voxels were resampled to isotropic 3 × 3 × 3 mm³ dimensions, and a Gaussian kernel with an 8-mm full width at half maximum (FWHM) was used for smoothing.

After preprocessing, volumes exhibiting scan-to-scan motion greater than 1.5 mm (Euclidean distance, radius = 65 mm) were repaired using the ArtRepair toolbox (Mazaika et al., 2007), employing linear interpolation between the nearest non-affected scans. Additionally, volumes with or without movement surrounded by scans with motion above 1.5 mm were flagged. Runs, where more than 20% of volumes were flagged, were excluded from analysis, leading to the exclusion of one child completing the match recognition task. When feasible, runs with excessive movement were repeated during the MR session. One binary regressor of no interest was included for each flagged volume to account for these movements (scrubbing).

In addition, the standard denoising pipeline (Nieto-Castanon, 2020) in CONN (Whitfield-Gabrieli & Nieto-Castanon, 2012) release 22.v2407 (Nieto-Castanon & Whitfield-Gabrieli, 2022) and SPM (W. Penny et al., 2007) release 12.7771 was used to obtain the potential confounding effects characterized by white matter time series (5 CompCor noise components), CSF time series (5 CompCor noise components), movement regressors and their first order derivatives (12 components), session and task effects and their first order derivatives (6 factors), and linear trends (2 factors) within each functional run, followed by bandpass frequency filtering of the BOLD time series (Hallquist et al., 2013) between 0.008 and 0.09 Hz. CompCor (Behzadi et al., 2007; Chai et al., 2012) noise components within white matter and CSF were estimated by computing the average BOLD signal as well as the largest principal components orthogonal to the BOLD average within each subject’s eroded segmentation masks. The 10 CompCor noise regressors for white matter and CSF were then added as regressors of no interest to the first-level GLMs.

### 2.4 Computational modelling of surprise and RPEs

#### 2.4.1 Statistical learning

To investigate statistical learning, we implemented the Shannon surprise model for both task versions to calculate trial-wise surprise values based on Bedi et al. (2025) and Konovalov and Krajbich (2018). In this model, we calculate the probability of the stimulus combination x of the current trial t:

$$p(x_{t})=\;\frac{1\;+\;\sum_{i=0}^{t-1}x_{i}}{n\;+\;\left(t-1\right)}.$$ (1)

This leads to $p(x_{0})=1\;/\text{​}n$ for the first trial. With n being the number of matching and non-matching stimulus pairs (n = 9 . for the match recognition task and n = 16 for the discriminative choice task; see Section 2.2). In the discriminative choice task, the stimulus combination x refers to the triplet combination of two visual and either one auditory or one tactile stimulus. In the match recognition task, x refers to the audio–visual and tactile–visual stimulus pairs. Then, the surprise value $I_{S,t}$ is obtained by calculating the negative logarithm of $p(x_{t})$ for each trial (Eq. 2):

$$I_{S,t}=\;-\log p(x_{t}).$$ (2)

For the discriminative choice task, we modified the calculation of the Shannon surprise to only account for the non-matching pair, since in this task version, the matching symbol is alwaypresented, and no surprise is, therefore, associated with this stimulus. The probability for the non-matching surprise was calculated using the same formulae as the regular surprise. But in this case, the stimulus combination x only refers to the non-matching audio–visual or tactile–visual stimulus pair. Here, we counted how often the non-matching pair was already presented and n=4 , because there are four possible non-matching pairs in each trial.

#### 2.4.2 Reinforcement learning

We used a Rescorla–Wagner model, a well-known model for reinforcement learning, to design the trial-wise updating mechanism for the learning process (Rescorla & Wagner, 1972). Based on the methodology described by Pedersen et al. (2017), a drift diffusion model (DDM; Ratcliff, 1978) was implemented to simulate basic multisensory processing involved in forming computational representations of the mental model and the choice-making processes that convert them into responses. We were able to estimate trial-wise parameters, inferred from the reaction times and accuracies, describing the learning process by implementing this generative model (Pedersen et al., 2017). This approach permitted the calculation of trial-wise values for each multisensory association, drift rates, and RPEs.

In this study, we implemented different variations of the RW model for each task. With the RW model, we were able to calculate the RPEs and update the values V of the multisensory associations for each trial. For a trial t, the RPE is calculated by the difference between the actual reward R and the value V (= expected reward):

$$RPE_{t}=(R_{t}-V_{t}).$$ (3)

Next, the RPE was weighted by the learning rate η and used to update the value V for the current trial t:

$$V_{t}^{C}=\;V_{t-1}^{C}+\;\eta^{C}\times \left(R_{t-1}-V_{t-1}^{C}\right).$$ (4)

The learning rate describes the extent to which values are updated after each trial (for a more detailed interpretation, see Zhang et al. (2020)). In this study, we implemented four different versions of the RW model for each task version, based on models proposed by Bedi et al. (2025), Frank et al. (2004), and Van Hamme & Wasserman (1994). In the first model (“simple RW”), only the value for the chosen response to the specific multisensory stimulus combination was updated. For example, in the discriminative choice task, the value for the left stimulus pair was updated after choosing the left ($V^{C}$). In the match recognition task, the value for the presented stimulus pair was updated. We also implemented the “asymmetric simple RW” model, where we used different learning rates for positive ($\eta_{+}^{C}$) and negative ($\eta_{-}^{C}$) feedback:

$$V_{t}^{C}=\{\begin{array}{c} V_{t-1}^{C}+\;\eta_{+}^{C}\times \left(R_{t-1}-V_{t-1}^{C}\right),\;\;if\;\;R=1\\ V_{t-1}^{C}+\;\eta_{-}^{C}\times \left(R_{t-1}-V_{t-1}^{C}\right),\;\;if\;\;R=0 \end{array}.$$ (5)

Next, we implemented the “transfer RW”, where additionally the value for the non-chosen or other pair ($V^{O})$ in the discriminative choice task and the values for all stimulus combinations containing one of the presented unisensory stimuli were updated. For example, in the match recognition task, visual stimulus 0 was presented with auditory/tactile stimulus A, therefore, all combinations either containing visual stimulus 0 or auditory/tactile stimulus A were also updated. Although these other combinations were only updated after positive feedback.

$$V_{t}^{O}=\;V_{t-1}^{O}+\;\eta^{O}\times \left(R_{t-1}-V_{t-1}^{O}\right).$$ (6)

Finally, we also implemented the “asymmetric transfer RW” model with different learning rates for positive $\eta_{+}^{C}$ and negative $\eta_{-}^{C}$ feedback. This resulted in four learning rates in the discriminative choice task (two for the chosen and two for the other pair) and three learning rates for the match recognition task since the other pairs were only updated after positive feedback.

To ensure that the sum of the values from both options did not exceed 1, we used a normalization method that reflects the joint evidence from both options, similar to odds ratio normalization (see Bogacz et al., 2006):

$${\bar{V}}_{t}^{C}=\frac{V_{t}^{C}\times \;\left(1-V_{t}^{O}\right)}{\left(V_{t}^{C}\;\times \left(1-V_{t}^{O}\right)+V_{t}^{O}\times \;\left(1-V_{t}^{C}\right)\right)},{\bar{V}}_{t}^{O}=1-{\bar{V}}_{t}^{C}.$$ (7)

Next, the values were used in the DDM to calculate the drift rate v. For the discriminative choice task, the difference between the two normalized values was multiplied by the drift weight $v_{mod}$ :

$$v_{t}=\;\left({\bar{V}}_{t}^{C}-{\bar{V}}_{t}^{O}\right)\;\;\times \;v_{mod}.$$ (8)

For the match recognition task, the drift rate was calculated as follows:

$$v_{t}=\;\left(V_{t}^{C}-\left(1-V_{t}^{C}\right)\right)\;\;\times \;v_{mod}.$$ (9)

This highlights that the drift weight can be seen as a scaling factor and as behavioural sensitivity to value differences (e.g., when there is a small difference between the two values, the drift rate will be high with a higher drift weight; Pedersen et al., 2017). The Wiener first passage time (WFPT) algorithm (Navarro & Fuss, 2009) was then used to fit the choice behaviour and reaction time data:

RT(x)~WFPT[a,τ,z,v(t)]. (10)

With this algorithm, the probability density for hitting both boundaries at any given time point t could be calculated:

$$\begin{array}{l} f\left(t\text{|}v,a,z\right)\\ \;\;=\frac{\pi }{a^{2}}\exp\left(-vaz-\frac{v^{2}t}{2}\right)\sum_{k=1}^{\infty }k\exp\left(-\frac{k^{2}\pi^{2}t}{2a^{2}}\right)\sin\left(k\pi z\right). \end{array}$$ (11)

**Fig. 2.**
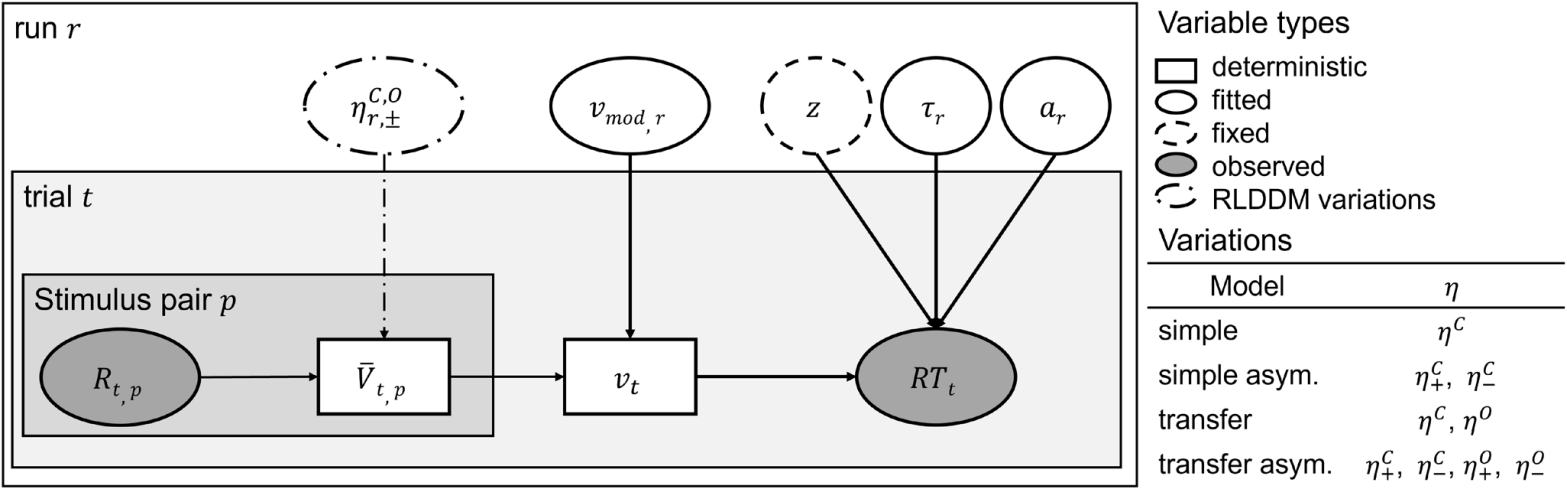
Schematic of the reinforcement learning drift diffusion model. *Note*. Deterministic variables are represented by rectangular nodes, fitted parameters by round nodes with solid lines, fixed parameters by round nodes with dashed lines, and observed data by grey round nodes. The parameters are estimated for each run separately. Learning rates η modulate the trial-wise values $\bar{V}$ which are updated depending on the feedback. The difference between the value of the left and right stimulus pair is multiplied by the $v_{mod}$ parameter to estimate the trial-wise drift rate v. Parameters are estimated for each run r. a = decision boundary; τ = non-decision time; z = starting point (fixed parameter); R = feedback; t = trial; p = audio–visual or tactile–visual stimulus pair; v = drift rate; RT = reaction time; r = run; C = chosen pair; O = other pair; $\eta_{+}$ = learning rate after positive feedback; $\eta_{-}$ = learning rate after negative feedback.

Finally, this returned the probability for choosing x given the value and the reaction time. Further details can be found in Pedersen et al. (2017). All used models are summarized in Figure 2.

For all the RW models, the learning rates were free parameters, and the initial values for all stimulus combinations were set to 0.5 (uninformed unbiased initial values). In the DDM, the free parameters were the non-decision time τ, the boundary separation a, and the drift weight $v_{mod}$ . We fixed the starting point to 0.5* a , which suggests no initial bias for one of the choices. The boundary separation reflects the amount of information needed to reach a decision threshold. Non-decision time accounts for the duration unrelated to the decision-making process, such as the time taken for motor execution. The drift rate represents the speed at which information accumulates toward a decision. In this study, trial-by-trial estimates were obtained for the RPEs, values, and drift rate, while a single value per run was estimated for the learning rate, boundary separation, and non-decision time.

To verify that all model parameters could be recovered successfully, we simulated data and performed parameter recovery analyses before fitting the models on the real data (see Supplementary Material S2). Next, the validated models were applied to the collected behavioural data for parameter estimation. Parameters were estimated independently of each subject and run. We then calculated the Bayesian information criterion (BIC) values (W. D. Penny et al., 2004; Schwarz, 2007) for each model, subject, and run, and selected the best-fitting model per run. The estimated parameters from the best-fitting model per run were then used in the statistical analyses and to link behavioural patterns to neural processes (Farrell & Lewandowsky, 2018; Wilson & Collins, 2019). This approach ensured that the models effectively captured the cognitive mechanisms underlying multisensory learning. The model fitting process was implemented in MATLAB using the Genetic Algorithm (GA) from the Global Optimization Toolbox, following the guidelines provided by Wilson and Collins (2019).

### 2.5 Statistical analyses

The statistical analyses were once done to examine the effects of age within the match recognition task. For this, we compared the data from the adults and the children who completed the match recognition task. Then, the analyses were repeated to examine the effect of the task version. Here, we compared the data from the two groups of children.

#### 2.5.1 Analyses of task performance and computational parameters

None of the included runs had more than 20% omissions. Moreover, as per the recommendations by Berger and Kiefer (2021), trials without a response, those with reaction times (RTs) under 200 ms, and those exceeding 3 standard deviations per run were removed from subsequent analyses. The excluded trials were also omitted from the RPE MR analyses.

We calculated the mean reaction time for correct trials, mean accuracy values (correct responses divided by the total number of trials, not counting omissions), and mean absolute drift rates for each bin per run to evaluate learning outcomes. One bin represented one-third of a run (14 trials per bin in the match recognition task; 15, 15, and 14 trials per bin in the discriminative choice task). We then used linear mixed models (LMMs) to analyse the effects of either age group or task version on the reaction time, accuracy, learning rate, non-decision time, boundary separation, drift weight, and absolute drift rate (library “lmerTest”, Kuznetsova et al., 2017). The models additionally included modality as a fixed effect to account for modality-specific differences (see Raduner et al., 2025). Bin was included as a fixed effect for the models investigating reaction times, accuracies, and absolute drift rate. For each analysis, we built the full linear mixed model with the interactions and main effects of all variables of interest as fixed effects (effects of age group: modality × bin × group; effects of task version: modality × bin × version) and the random intercept for subject (1 | subject). To investigate the effect of surprise on the reaction time, we built the same full linear mixed models as described above without the effect of bin and added the surprise as a fixed effect. Here, we used the trial-wise reaction times and surprise values. Then we used the step function (library “lmerTest”, Kuznetsova et al., 2017) on the full model to remove all effects that do not significantly improve model fit. We added gender to all the LLMs to account for the unequal gender distributions between the groups. We conducted post hoc analyses adjusting *p*-values by the Tukey method to correct for multiple comparisons (library “emmeans,” Lenth, 2024) for significant main effects with more than one level. The R Statistical language (version 4.4.1; R Core Team, 2024) was used for the analyses.

#### 2.5.2 Whole-brain analyses

For the first-level analysis, we used a general linear model (GLM) that included the individual onsets of two main regressors: stimulus and feedback onsets, along with two parametric modulators: surprise and prediction error. The surprise modulator was added to the stimulus regressor, while the prediction error modulator was added to the feedback regressor. The GLM also included two additional regressors of no interest: one with feedback onsets for neutral trials and one with feedback onsets for RT outliers. Since statistical learning is thought to be an implicit process, we did not exclude trials with no response or RT outliers for the surprise analyses. Additionally, each model included six regressors for the six realignment parameters and an extra regressor of no interest for each flagged scan, when applicable. Finally, we added the 10 CompCor regressors as an additional noise correction. The GLMs included the first AV and TV run for each participant with sufficient data quality and were convolved with the canonical haemodynamic response function as implemented in SPM12.

Whole-brain second-level two-sample t-tests were conducted to examine brain activation modulated by surprise and RPEs for AV and TV runs combined and the differences between the adults and children and task versions. All the second-level analyses also incorporated gender and handedness as covariates. With these analyses, we aimed to investigate brain networks involved in statistical and reinforcement learning and how these are affected by age group and task design. We use a voxel-wise threshold of *p* < .005 (uncorrected) to threshold statistical parametric maps and cluster correction for multiple comparisons with the family wise error (FWE) rate of *p* < .05.

#### 2.5.3 Regions of interest (ROI) analyses

**Fig. 3.**
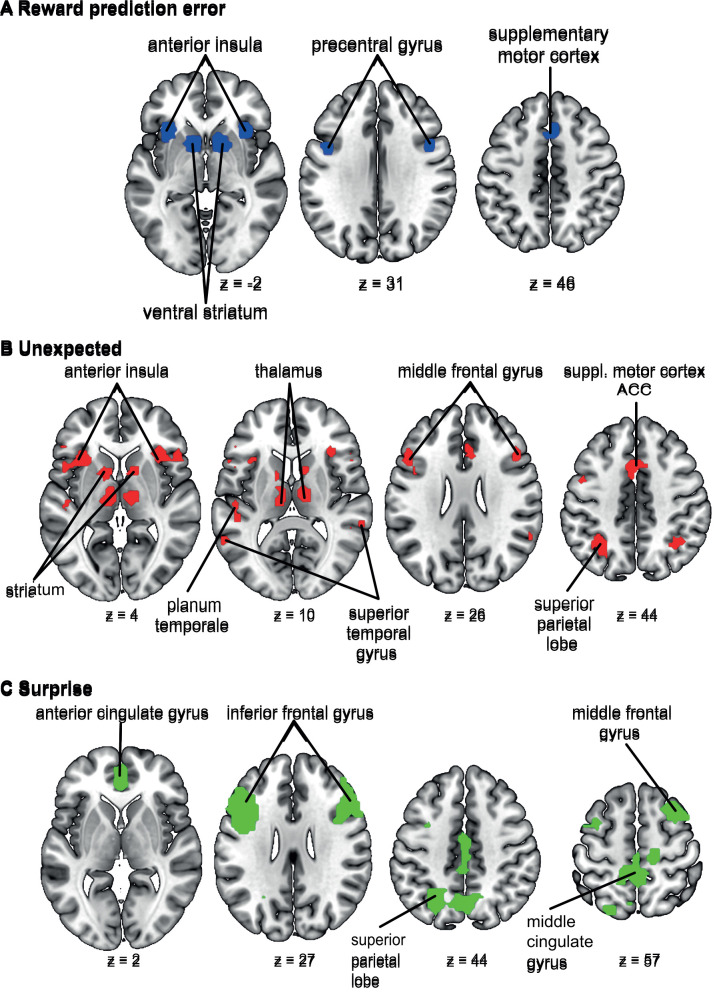
Depiction of masks used for ROI analyses. (A) The clusters included in the RPE networks from Neurosynth. (B) The network related to the term “unexpected” from Neurosynth and (C) the clusters from the surprise analyses described in Section 3.3.1.

To investigate the effects of age group and task version in more detail, we examined brain activation in ROIs related to surprise signals and RPEs. For this, we used the same RPE network ROI as in Raduner et al. (2025). This ROI was generated with www.neurosynth.org using the term “prediction error” (see Raduner et al. (2025) for more details). Additionally, we also used the term “unexpected” on neurosynth for a network related to surprise processing (Modirshanechi et al., 2023). These networks were used to investigate modality and age group effects on neural reward prediction error and surprise processing. After downloading, the networks were binarized using SPM12 and the distinct clusters were extracted using MarsBaR. To investigate the effects of the task version on surprise and reward prediction errors, we used the clusters from the whole brain analyses of the adults and children completing the match recognition tasks and the reward prediction error network from neurosynth. Figure 3 displays the masks and corresponding sub-regions utilized for these analyses.

MarsBaR was utilized to derive the mean beta values from the regions of interest (ROIs). By employing the code available through MarsBaR (https://marsbar-toolbox.github.io/faq.html, we successfully extracted beta values that were adjusted for the mean signal within each ROI (Pernet, 2014). These beta values reflect the percentage of signal change within the specified ROI. We computed the mean beta separately for AV and TV runs for each subcluster of each network. In the surprise network ROI, we extracted the beta values from the surprise regressor, and in the RPE network ROI from the RPE regressor. Subsequently, these beta values were analysed using linear mixed models in R to explore the differences between adults and children and task versions. The LMM for the age group comparison included the interaction between group and modality, and the interaction between task version and modality to investigate the effects of task version. Gender was added as a covariate to all LMMs. To address the issue of multiple comparisons within one network ROI, all *p*-values derived from each linear mixed model were extracted and adjusted using the False Discovery Rate (FDR) method (Benjamini & Hochberg, 1995). The FDR correction was implemented with a significance threshold of α=0.05 , enabling us to manage the proportion of false positives among the results.

## 3 Results

In this section, key findings from the analyses are outlined. We first compared behavioural, computational, and neural data between the adult and children group, which completed the match recognition task, and then between the two groups of children to compare the two task versions.

### 3.1 Task performance

To provide a comprehensive view of task performance, we first examined how reaction times and accuracy varied across sensory modalities (AV vs. TV), trial progression (early, middle, late bins), and participant characteristics (age group or task version).

#### 3.1.1 Age-related effects in the match recognition task (adults vs. children)

##### 3.1.1.1 Reaction time

**Fig. 4.**
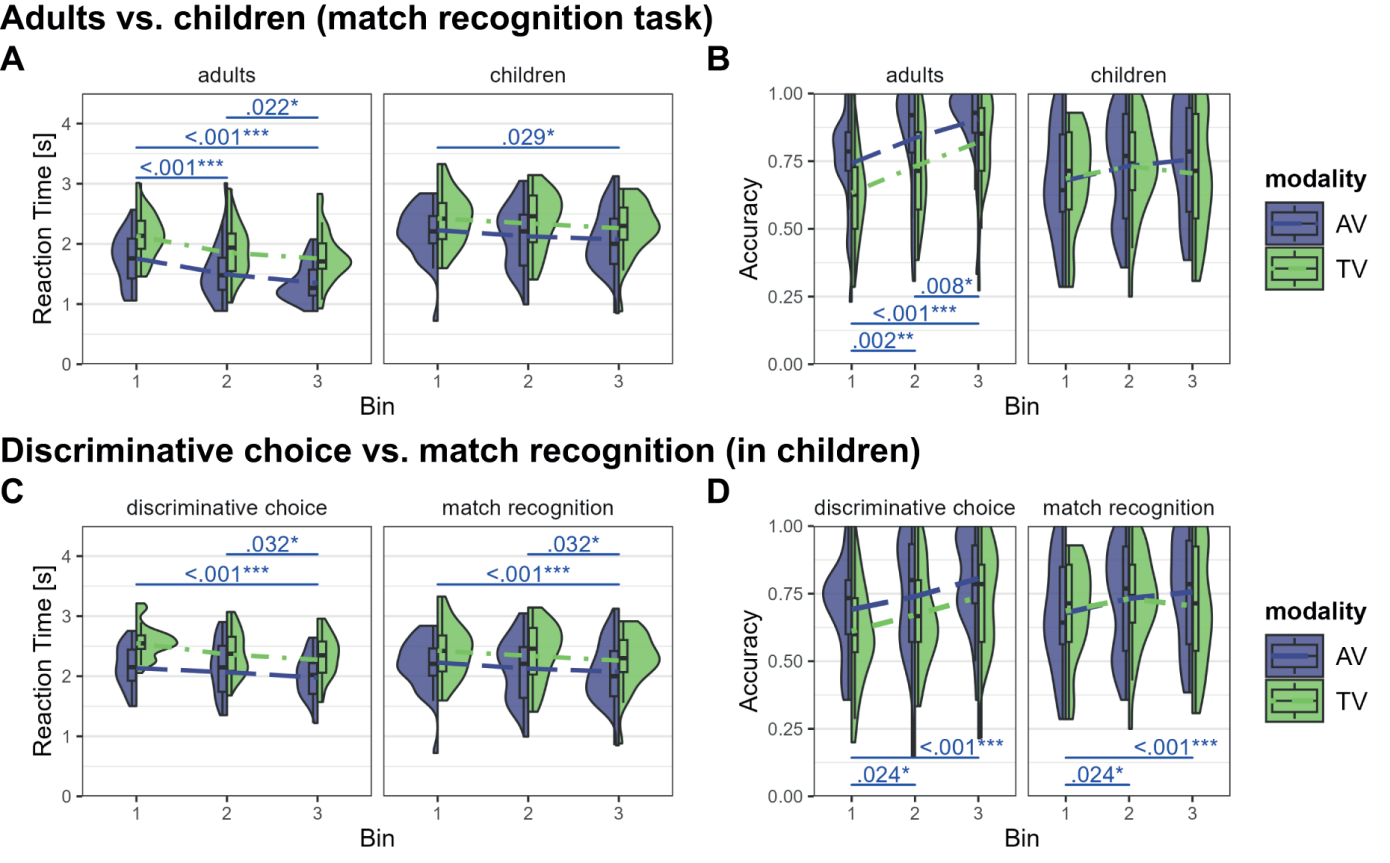
Reaction time and accuracy per modality (audio–visual vs. tactile–visual), group (adults vs. children), task version (discriminative choice vs. match recognition), and bin. (A) The reaction times and (B) the accuracies per task and bin on the x-axis for the comparison between adults and children. (C) The reaction times and (D) the accuracies per task and bins for the comparison between the discriminative choice and match recognition task in children. AV = audio–visual (blue), TV = tactile–visual (green), bin1 = 1^st^ third of trials, bin2 = 2^nd^ third of trials, bin3 = last third of trials.

The best-fitting LMM for reaction time included fixed effects of group, modality, bin, and gender, as well as the interactions between group and modality and group and bin, and a random intercept per participant. It showed a significant main effect of group (*F*(1,53) = 26.57, *p* < .001; η^2^_p_ = .33, 95% CI [.17, 1.00]), modality (*F*(1,274) = 97.29, *p* < .001; η^2^_p_ = .26, 95% CI [.19, 1.00]), and bin (*F*(2,274) = 22.05, *p* < .001; η^2^_p_ = .14, 95% CI [.08, 1.00]), and a significant interaction between group and modality (*F*(1,274) = 5.61, *p* = .019; η^2^_p_ = .02, 95% CI [.03, 1.00]) and between group and bin (*F*(2,274) = 4.80, *p* = .009; η^2^_p_ = .03, 95% CI [.005, 1.00]). Post hoc pairwise comparisons between the bins and modality are given in Table 4. The results showed faster reaction times for adults than for children. While for adults, reaction times differed significantly between all bins, for children, only the difference in reaction time between the first and last bin was significant. This indicates faster reaction times over time within a run. We also observed faster reaction times for AV runs than for TV runs with a weaker effect in children (see Fig. 4A).

**Table 4.** Post hoc tests of the interaction effects between group (adults vs. children) and bin, and group and modality on reaction time and accuracy.

Model		Contrast	Estimate	*SE*	*df*	*t*	*p*
reaction time ~ bin | group	adults	**bin1 - bin2**	**0.23**	**0.05**	**274.00**	**4.14**	<.001***
		**bin1 - bin3**	**0.37**	**0.05**	**274.00**	**6.81**	<.001***
		**bin2 - bin3**	**0.15**	**0.05**	**274.00**	**2.66**	.022*
	children	bin1 - bin2	0.06	0.05	274.00	1.06	.538
		**bin1 - bin3**	**0.14**	**0.05**	**274.00**	**2.57**	.029*
		bin2 - bin3	0.08	0.05	274.00	1.50	.291
reaction time ~ group | bin	bin1	**A – C**	**-0.41**	**0.11**	**73.05**	**-3.59**	.001***
	bin2	**A – C**	**-0.58**	**0.11**	**73.05**	**-5.05**	<.001***
	bin3	**A – C**	**-0.64**	**0.11**	**73.05**	**-5.61**	<.001***
reaction time ~ modality | group	adults	**AV - TV**	**-0.38**	**0.04**	**274.00**	**-8.65**	<.001***
	children	**AV - TV**	**-0.24**	**0.04**	**274.00**	**-5.30**	<.001***
reaction time ~ group | modality	AV	**A – C**	**-0.62**	**0.11**	**62.74**	**-5.62**	<.001***
	TV	**A – C**	**-0.47**	**0.11**	**62.74**	**-4.26**	<.001***
accuracy ~ bin | group	adults	**bin1 - bin2**	**-0.09**	**0.03**	**274.00**	**-3.39**	.002**
		**bin1 - bin3**	**-0.17**	**0.03**	**274.00**	**-6.42**	<.001***
		**bin2 - bin3**	**-0.08**	**0.03**	**274.00**	**-3.03**	.008**
	children	bin1 - bin2	-0.04	0.03	274.00	-1.68	.215
		bin1 - bin3	-0.05	0.03	274.00	-1.81	.169
		bin2 - bin3	-0.00	0.03	274.00	-0.13	.991
accuracy ~ group | bin	bin1	A – C	-0.01	0.05	87.31	-0.17	.865
	bin2	A – C	0.04	0.05	87.31	0.83	.411
	bin3	**A – C**	**0.12**	**0.05**	**87.31**	**2.52**	.014*
accuracy ~ modality | group	adults	**AV - TV**	**0.10**	**0.02**	**274.00**	**4.61**	<.001***
	children	AV - TV	0.02	0.02	274.00	0.73	.465
accuracy ~ group | modality	AV	**A – C**	**0.09**	**0.04**	**69.45**	**2.11**	.039*
	TV	A – C	0.01	0.04	69.45	0.14	.889

*Note*. Degrees-of-freedom method: Kenward–Roger. *P* value adjustment: Tukey method. Significant post hoc differences in bold. *p < .05, **p ≤ .01, ***p ≤ .001. AV = audio–visual, TV = tactile–visual, A = adults, C = children, bin1 = 1^st^ third of trials, bin2 = 2^nd^ third of trials, bin3 = last third of trials.

##### 3.1.1.2 Accuracy

The best-fitting LLM for accuracy included fixed effects of group, modality, bin, and gender, as well as the interactions between group and modality and group and bin, and a random intercept per participant. It showed a significant main effect of modality (*F*(1,274) = 14.29, *p* < .001; η^2^_p_ = .05, 95% CI [.02, 1.00]) and bin (*F*(2,274) = 17.21, *p* < .001; η^2^_p_ = .11, 95% CI [.06, 1.00]). The effect of group is not significant (*F*(1,53) = 1.45, *p* = 0.234; η^2^_p_ = .03, 95% CI [.00, 1.00]). There was also a significant interaction between group and modality (*F*(1,274) = 7.54, *p* = .006*;* η^2^_p_ = .03, 95% CI [.004, 1.00]), and between group and bin (*F*(2,274) = 5.43, *p* = .005*;* η^2^_p_ = .04, 95% CI [.007, 1.00]). Post hoc pairwise comparisons between the bins and modality are given in Table 4. These results indicate that accuracies increase over time within runs and are higher for AV runs than for TV runs. These effects are only significant in the adult group and not in the children’s group (see Fig. 4B).

#### 3.1.2 Task-related effects in children (match recognition vs. discriminative choice)

##### 3.1.2.1 Reaction times

The best-fitting LMM for reaction time included fixed effects of modality, bin and gender, no interactions, and a random intercept per participant. It showed a significant main effect of modality (*F*(1,282) = 86.15, *p* < .001; η^2^_p_ = .23, 95% CI [.17, 1.00]) and bin (*F*(2,282) = 10.81, *p* < .001; η^2^_p_ = .07, 95% CI [.03, 1.00]) and no effect of gender (*F*(1,55) = 0.06, *p* = .800; η^2^_p_ = .001, 95% CI [.00, 1.00]). Post hoc tests are given in Table 5. These results indicate faster reaction times in the last third within runs than the first and second thirds. Within both task versions, reaction times were faster for AV than for TV runs with no differences between task versions (see Fig. 4C).

**Table 5.** Post hoc tests of the main effects of bin and modality on reaction time and accuracy.

Model	Contrast	Estimate	SE	*df*	*t*	*p*
reaction time ~ bin	bin1 - bin2	0.08	0.04	282.00	2.12	.088
	**bin1 - bin3**	**0.17**	**0.04**	**282.00**	**4.64**	<.001***
	**bin2 - bin3**	**0.09**	**0.04**	**282.00**	**2.53**	.032*
reaction time ~ modality	**AV - TV**	**-0.28**	**0.03**	**282.00**	**-9.28**	<.001***
accuracy ~ bin	**bin1 - bin2**	**-0.05**	**0.02**	**282.00**	**-2.64**	.024*
	**bin1 - bin3**	**-0.09**	**0.02**	**282.00**	**-4.49**	<.001***
	bin2 - bin3	-0.04	0.02	282.00	-1.85	.156
accuracy ~ modality	**AV - TV**	**0.04**	**0.02**	**282.00**	**2.83**	.005**

*Note*. Degrees-of-freedom method: Kenward–Roger. *P* value adjustment: Tukey method. Significant post hoc differences in bold. *p < .05, **p ≤ .01, ***p ≤ .001. AV = audio–visual, TV = tactile–visual, bin1 = 1^st^ third of trials, bin2 = 2^nd^ third of trials, bin3 = last third of trials.

##### 3.1.2.2 Accuracy

The best-fitting LMM for accuracy included fixed effects of modality, bin and gender, no interactions, and a random intercept per participant. It showed significant main effects of modality (*F*(1,282) = 8.02, *p* = .005; η^2^_p_ = .03, 95% CI [.005, 1.00]) and bin (*F*(2,282) = 10.18, *p* < .001*;* η^2^_p_ = .07, 95% CI [.03, 1.00]) and no main effect of gender (*F*(1,55) = 0.24, *p* = .629; η^2^_p_ = .004, 95% CI [.00, 1.00]). Post hoc tests are given in Table 5. The results indicate increasing accuracies over time within runs in both tasks with no differences between the two task versions, and higher accuracies AV than TV runs (see Fig. 4D).

### 3.2 Computational modelling

To explore how decision making and learning parameters varied across conditions, we examined differences in learning rate, non-decision time, drift weight, and boundary separation across sensory modalities and participant groups. For the drift rate, we additionally considered changes across trial progression to capture temporal dynamics in evidence accumulation. The parameters from the best-fitting model for each subject and run were used for these analyses. The mean parameter values per modality, group, and task version are summarized in Table 6. These descriptive values are provided to illustrate general trends in the data and to contextualize the subsequent statistical analyses.

**Table 6.** Mean values and standard deviation for each model parameter per modality and task version.

	Match recognition				Discriminative choice	
	Adults		Children		Children	
	AV	TV	AV	TV	AV	TV
Learning rate	0.35 (0.28)	0.23 (0.25)	0.38 (0.33)	0.38 (0.36)	0.33 (0.34)	0.36 (0.40)
Non-decision time	0.69 (0.20)	0.92 (0.45)	0.89 (0.44)	1.12 (0.45)	0.90 (0.30)	1.14 (0.37)
Drift weight	3.60 (3.30)	4.21 (3.50)	1.96 (2.26)	1.10 (0.93)	2.42 (3.27)	2.29 (3.47)
Decision boundary	2.32 (0.41)	2.28 (0.42)	2.48 (0.42)	2.46 (0.38)	2.48 (0.26)	2.59 (0.58)
Drift rate [bin 1]	0.52 (0.42)	0.22 (0.22)	0.28 (0.32)	0.20 (0.25)	0.30 (0.29)	0.22 (0.25)
Drift rate [bin 2]	1.15 (0.70)	0.73 (0.65)	0.53 (0.43)	0.46 (0.45)	0.64 (0.52)	0.46 (0.40)
Drift rate [bin 3]	1.46 (0.74)	1.01 (0.81)	0.64 (0.46)	0.57 (0.51)	0.81 (0.62)	0.57 (0.46)

*Note*. The mean values for each parameter from the RLDDM model are displayed for all groups and task versions. AV = audio–visual, TV = tactile–visual, bin 1 = 1^st^ third of trials within run, bin 2 = 2^nd^ third of trials within run, bin 3 = last third of trials within run.

#### 3.2.1 Age-related effects in the match recognition task (adults vs. children)

##### 3.2.1.1 Surprise

**Fig. 5.**
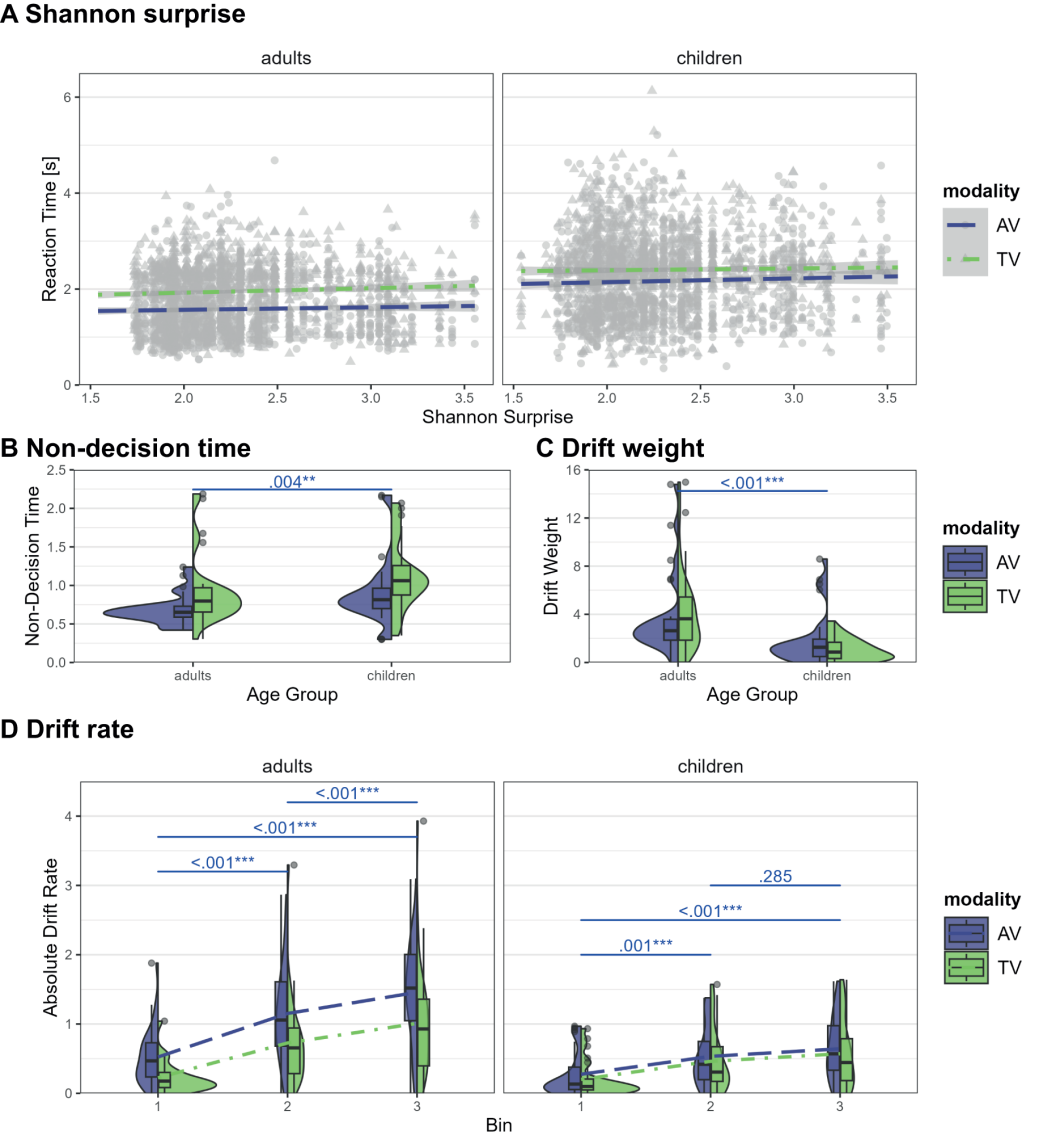
Effects of surprise, modality, and group on reaction time and RLDDM parameters. (A) The effect of surprise on reaction time. (B) The non-decision time across modality and group. (C) The drift weight across modality and group. (D) The drift rate as a function of modality, group, and bin. Error bars represent standard errors of the mean. AV = audio–visual in blue, TV = tactile–visual in green, bin1 = 1^st^ third of trials, bin2 = 2^nd^ third of trials, bin3 = last third of trials.

The LMM with the main effects of surprise, group, modality, and gender, the interaction between modality and group, and a random intercept per participant best described the reaction time data. The main effects of surprise (*F*(1,4453.2) = 8.16, *p* = .004*;* η^2^_p_ = .002, 95% CI [.0003, 1.00]), modality (*F*(1,4453.2) = 287.09, *p* < .001*;* η^2^_p_ = .06, 95% CI [.05, 1.00]), group (*F*(1,53.0) = 25.77, *p* < .001*;* η^2^_p_ = .33, 95% CI [.16, 1.00]), and the interaction between modality and group (*F*(1,4453.2) = 16.27, *p* < .001*;* η^2^_p_ = .004, 95% CI [.001, 1.00]) were statistically significant. These results show slower reaction times for more surprising trials (see Fig. 5A; among the other effects described in Section 3.1).

##### 3.2.1.2 Model fitting

The model selection results are displayed in Table 7. In both groups, the simple RW was overall the best-fitting model, but in AV runs for 15 of 28 participants, the transfer RW was the best-fitting model, whereas in TV runs, it was only for 8 of 28 participants. A two-sample test for equality of proportions with Yates’ continuity correction revealed a significant difference in the proportions of participants selecting the transfer RW model between the simple RW and the transfer RW model in the children group, $\chi^{2}$(1) = 30.04, *p* < .001. Significantly more children had a variation of the simple RW as the best-fitting model (76.79%), compared with 23.21% having a variation of the transfer RW model as the best-fitting model. In the adult group, there was no significant difference (58.93% vs. 41.07% having a variation of the simple RW model compared with the transfer RW model, respectively; $\chi^{2}$(1) = 2.89, *p* = .089). These results suggest that children employ simpler learning strategies during the MSL task than adults.

**Table 7.** Model selection results for adults and children completing the match recognition task.

	Adult group		Children group	
Model	AV	TV	AV	*TV*
Simple RW	11	15	15	21
Simple asymmetric RW	2	5	5	2
Transfer RW	11	5	8	3
Transfer asymmetric RW	4	3	0	2

*Note*. The table shows the number of subjects for whom each model was the best fitting, categorized by modality (auditory vs. tactile). The values represent the count of subjects for which each model provided the best fit in each condition. AV = audio–visual, TV = tactile–visual, RW = Rescorla–Wagner.

##### 3.2.1.3 Reinforcement learning

We fit a full LMM with effects of modality, group, and the interaction between the two. For the absolute drift rate, we additionally added bin to the interaction term. We then used the step function to determine the best-fitting model for each parameter and added gender as a covariate. The best-fitting LMM for the learning rate and the decision boundary only included the main effect of gender, with no interaction. The best-fitting LMM for the non-decision time included the main effect of modality, group, and gender, no interactions, and a random intercept per participant. The best-fitting LMM for the drift weight included group and gender as main effects, no interactions, and a random intercept per participant. The best-fitting LMM for the drift rate included the main effect of group, modality, bin and gender, the interaction between modality and group and bin and group, and a random intercept per participant. We observed a significant main effect of group on the non-decision time (*F*(1,53) = 9.33, *p* = .004*;* η^2^_p_ = .15, 95% CI [.03, 1.00]), the drift weight (*F*(1,109) = 21.35, *p* < .001; η^2^_p_ = .16, 95% CI [.07, 1.00]), and the drift rate (*F*(1,53) = 9.80, *p* = .003; η^2^_p_ = .16, 95% CI [.04, 1.00]). Adding the main effect of group to the LMM for the learning rate showed a trend for the main effect of group on the learning rate (*F*(1,109) = 3.83, *p* = .053; η^2^_p_ = .03, 95% CI [.00, 1.00]). Additionally, we observed a significant interaction between modality and group (*F*(1,274) = 14.75, *p* < .001; η^2^_p_ = .05, 95% CI [.02, 1.00]) and group and bin (*F*(2,274) = 11.90, *p* < .001; η^2^_p_ = .08, 95% CI [.03, 1.00]) on the drift rate. The main effect of modality was significant for the non-decision time (*F*(1,55) = 23.04, *p* < .001; η^2^_p_ = .30, 95% CI [.14, 1.00]) and drift rate (*F*(1,274) = 31.25, *p* < .001; η^2^_p_ = .10, 95% CI [.05, 1.00]). For the drift rate, the main effect of bin was also significant (*F*(2,274) = 75.97, *p* < .001; η^2^_p_ = .36, 95% CI [.28, 1.00]). The main effect of gender was significant for the non-decision time (*F*(1,53) = 5.96, *p* = .018; η^2^_p_ = .10, 95% CI [.01, 1.00]). These results indicate that the adults showed shorter non-decision times (see Fig. 5B), higher drift weights (see Fig. 5C), and drift rates (see Fig. 5D), suggesting that adults are more sensitive to smaller differences between values and process information faster. The drift rates increased within runs across all bins for adults, whereas in children, the drift rate only increased from the first to the second and third bin. Additionally, tactile–visual runs showed longer non-decision times and lower drift rates, but only in adults. Next, there is a trend for lower learning in adults than in children, indicating that children might rely more on recent outcomes, whereas adults tend to update information more conservatively. Lastly, male participants showed faster non-decision times. Table 8 gives the post hoc tests for the interaction effects on the drift rate.

**Table 8.** Post hoc tests of the interaction effects between bin and group and modality and group on the drift rate.

Model		Contrast	Estimate	*SE*	*df*	*t*	*p*
Drift rate ~ bin | group	adults	**bin1 - bin2**	**-0.56**	**0.07**	**274.00**	**-7.87**	<.001***
		**bin1 - bin3**	**-0.86**	**0.07**	**274.00**	**-11.97**	<.001***
		**bin2 - bin3**	**-0.29**	**0.07**	**274.00**	**-4.10**	<.001***
	children	**bin1 - bin2**	**-0.26**	**0.07**	**274.00**	**-3.62**	.001**
		**bin1 - bin3**	**-0.37**	**0.07**	**274.00**	**-5.13**	<.001***
		bin2 - bin3	-0.11	0.07	274.00	-1.52	.285
Drift rate ~ group | bin	bin1	A - C	0.10	0.13	82.20	0.76	.447
	bin2	**A - C**	**0.40**	**0.13**	**82.20**	**3.10**	.003**
	bin3	**A - C**	**0.59**	**0.13**	**82.20**	**4.52**	<.001***
Drift rate ~	adults	**AV - TV**	**0.39**	**0.06**	**274.00**	**6.67**	<.001***
modality | group	children	AV - TV	0.07	0.06	274.00	1.24	.217
Drift rate ~	AV	**A - C**	**0.52**	**0.12**	**67.05**	**4.24**	<.001***
Group | modality	TV	A - C	0.21	0.12	67.05	1.66	.101

*Note*. Degrees-of-freedom method: Kenward–Roger. *P* value adjustment: Tukey method. Significant post hoc differences in bold. **p ≤ .01, ***p ≤ .001. A = adults, C = children.

#### 3.2.2 Task-related effects in children (match recognition vs. discriminative choice)

##### 3.2.2.1 Surprise

**Fig. 6.**
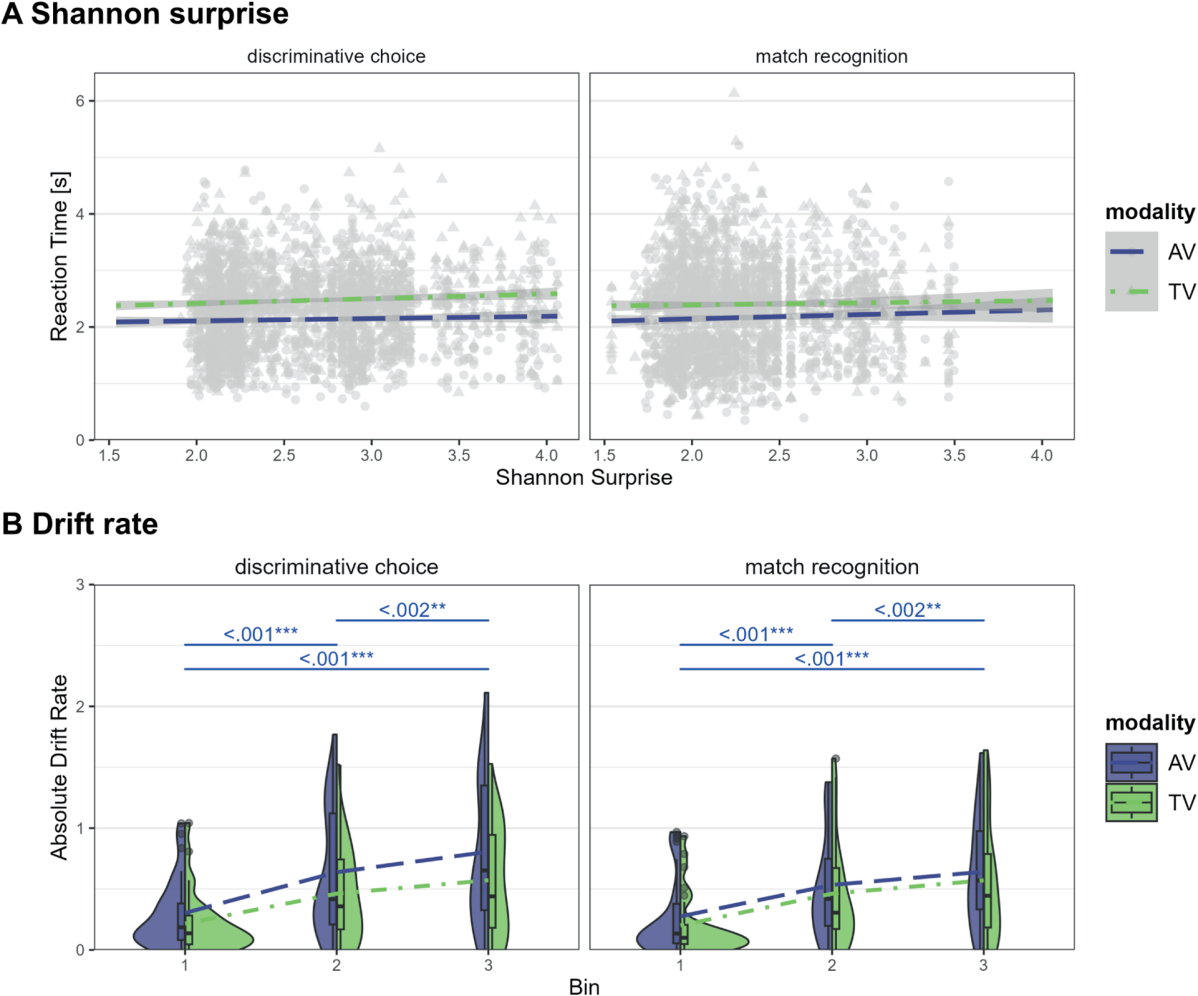
Effects of surprise, modality, and task version on reaction time and effects of modality and task version on the drift rate. (A) The reaction time as a function of Shannon surprise per task version (discriminative choice or match recognition task) and modality (audio–visual or tactile–visual). (B) The drift rate as a function of task version (discriminative choice or match recognition task) and modality (audio–visual or tactile–visual), and bin (1^st^, 2^nd^, and last third of trials within run). AV = audio–visual in blue, TV = tactile–visual in green.

The linear mixed model with the main effects of surprise, version, modality, the interaction effects between version and modality, and a random intercept per participant best described the reaction time data. The main effects of surprise (*F*(1,4687.1) = 8.52, *p* = .004; η^2^_p_ = .002, 95% CI [.0003, 1.00]), modality (*F*(1,4687.1) = 225.46, *p* < .001; η^2^_p_ = .05, 95% CI [.04, 1.00]), and the interaction effect between modality and version (*F*(1,4687.1) = 6.93, *p* = .009; η^2^_p_ = .001, 95% CI [.0002, 1.00]) were statistically significant. The main effect of version was not significant (*F*(1) = 0.01, *p* = .927; η^2^_p_ = .0002, 95% CI [.00, 1.00]). These results show slower reaction times for more surprising trials (see Fig. 6A; among the effects already described in Section 3.1). We also investigated the effect of the non-matching surprise on the reaction time in the discriminative choice task. Here, we did not observe any effect of the non-matching surprise on the reaction time (*F*(1, 2447.08) = 0.41, *p* = .522; η^2^_p_ = .0002, 95% CI [.00, 1.00]), therefore, we only investigated the Shannon surprise on a neural level.

##### 3.2.2.2 Model fitting

The model selection results are displayed in Table 9. For both task versions, the simple RW was overall the best-fitting model independent of modality. Significantly more children had a variation of the simple RW as the best-fitting model compared with a variation of the transfer RW model in the discriminative choice task (89.66% and 10.34%, respectively; *p* < .001) and the match recognition task (76.79% and 23.21%, respectively; *p* < .001). This suggests that across all task versions, children most often used simpler learning strategies during the MSL task.

**Table 9.** Model selection results for children either completing the discriminative choice or the match recognition task.

	Discriminative choice		Match recognition	
Model	AV	TV	AV	TV
Simple RW	24	18	15	21
Simple asymmetric RW	3	7	5	2
Transfer RW	2	3	8	3
Transfer asymmetric RW	0	1	0	2

*Note*. The table gives the number of subjects for whom each model was the best-fitting, categorized by model type (auditory vs. tactile). The values represent the count of subjects for which each model provided the best fit in each condition. AV = audio–visual, TV = tactile–visual, RW = Rescorla–Wagner.

##### 3.2.2.3 Reinforcement learning

We fit a full LMM with effects of modality, task version, and the interaction between the two. For the absolute drift rate, we additionally added bin to the interaction term. We then used the step function to determine the best-fitting model for each parameter and added gender as a covariate. The best-fitting LMM for the learning rate, decision boundary, and drift weight only included the main effect of gender, no interaction. The best-fitting LMM for the non-decision time included the main effect of modality and gender, with no interactions, and a random intercept per participant. The best-fitting LMM for the drift rate included the main effect of modality, bin and gender, no interactions, and a random intercept per participant. We did not observe any significant effect of task version on any of the modelling parameters, indicating no differences between information accumulation, value sensitivity, non-decision time and decision boundary between the two task versions. The main effect of bin (*F*(2,282) = 64.82, *p* < .001; η^2^_p_ = .31, 95% CI [.24, 1.00]) was significant for the drift rate which significantly increased from each third to the next (see Table 10 and Fig. 6B). The main effect of modality was significant for the non-decision time (*F*(1,56) = 28.99, *p* < .001; η^2^_p_ = .34, 95% CI [.18, 1.00]) and drift rate (*F*(1,282) = 16.80, *p* < .001; η^2^_p_ = .06, 95% CI [.02, 1.00]; see Fig. 6B), indicating that for TV runs compared with AV runs, non-decision times were longer and drift rates lower. We observed wider boundaries in male participants than in female participants (*F*(1,55) = 4.97, *p* = .030; η^2^_p_ = .08, 95% CI [.005, 1.00]) and a trend for shorter non-decision times in male participants (*F*(1,55) = 2.96, *p* = .091; η^2^_p_ = .05, 95% CI [.00, 1.00]). We did not observe any significant effects on the learning rate or the drift weight.

**Table 10.** Post hoc tests of the effect of bin on the absolute drift rate.

Model	Contrast	Estimate	*SE*	*df*	*t*	*p*
Drift rate ~ bin	**bin1 - bin2**	**-0.28**	**0.04**	**282.00**	**-7.67**	<.001***
	**bin1 - bin3**	**-0.40**	**0.04**	**282.00**	**-11.12**	<.001***
	**bin2 - bin3**	**-0.12**	**0.04**	**282.00**	**-3.45**	.002**

*Note*. Degrees-of-freedom method: Kenward–Roger. *P* value adjustment: Tukey method. Significant post hoc differences in bold. **p ≤ .01, ***p ≤ .001. bin1 = 1^st^ third of trials, bin2 = 2^nd^ third of trials, bin3 = last third of trials.

### 3.3 Modulation of the BOLD signal by model parameters

We performed whole brain analyses to investigate the neural differences between the adults and children and the two task versions related to reward prediction error and surprise processing. The trial-wise surprise values were used as a parametric modulator during stimulus processing, and the trial-wise reward prediction error values as a parametric modulator during feedback processing.

#### 3.3.1 Age-related effects in the match recognition task (adults vs. children)

##### 3.3.1.1 Surprise

**Fig. 7.**
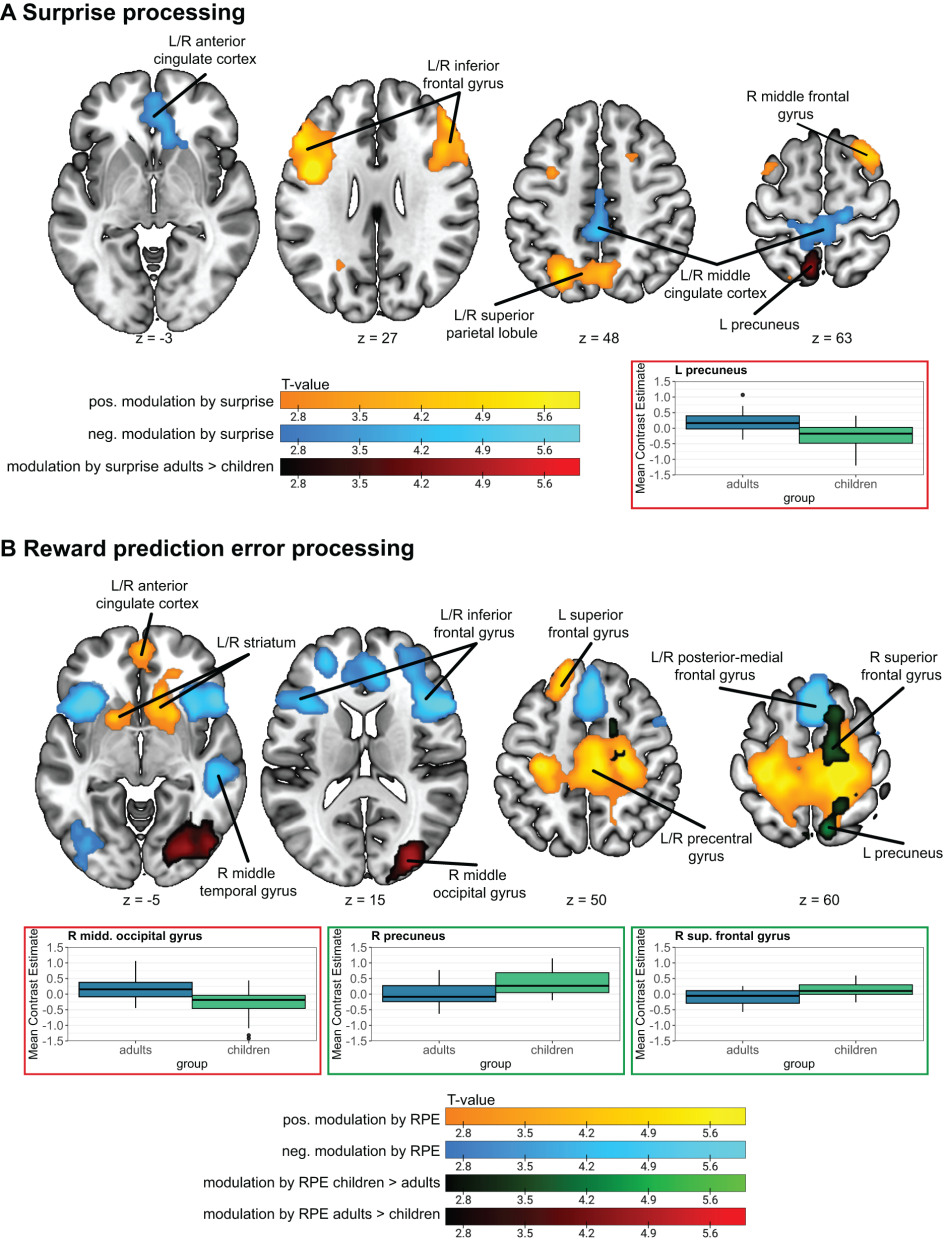
Age effects on the brain activation during stimulus and feedback processing modulated by surprise and RPE, respectively, in the match recognition task. The upper panel (A) shows the positive and negative modulation of the BOLD signal by the surprise parameter for both groups combined and the differences in the surprise modulation between adults and children (in red). (B) The positive and negative modulation of the BOLD signal by the reward prediction error parameter for both groups combined and the differences in the RPE modulation between children and adults (in green and red). Orange-yellow shows the positive modulation, light blue the negative modulation by the parameters. The boxplots show the mean extracted, whitened y values from SPM12 in the clusters where we found a significant difference between the two age groups. Adults in blue, children in green. Cluster defining threshold *p* < .005 (unc.), *p*_FWEc_ = .05, family error-wise corrected. Pos. = positive, neg. = negative, midd. = middle, sup. = superior, RPE = reward prediction error, R = right, L = left.

The surprise signal positively modulated brain activity in the left inferior frontal gyrus (pars opercularis), left superior parietal lobule, right inferior frontal gyrus (pars triangularis), and the right middle frontal gyrus for both groups combined. Brain activation across groups in the left middle and right anterior cingulate cortex was negatively modulated by surprise. Adults showed a stronger surprise signal in the left precuneus than the children. The opposite contrast did not show any differences (see Table 11 and Fig. 7A).

**Table 11.** Significant clusters modulated by surprise and RPE signals.

		MNI coordinates						
Contrast	Brain area	x	y	z	Cluster size	T-value	Peak-level *p*_unc_	Cluster-level *p*_FWE_
**Surprise**	L Inferior Frontal Gyrus (p. Opercularis)	-44	6	27	806	6.81	<.001	<.001
	L Superior Parietal Lobule	-26	-66	48	678	6.08	<.001	<.001
	R Inferior Frontal Gyrus (p. Triangularis)	52	36	24	440	5.77	<.001	<.001
	R Middle Frontal Gyrus	34	12	60	177	5.35	<.001	.032
	L Middle Cingulate Cortex	-2	-39	51	686	-5.25	<.001	<.001
	R Anterior Cingulate Cortex	10	30	-3	355	-4.13	<.001	.001
**adults** **>** **children**	L Precuneus	-8	-63	63	199	4.84	<.001	.019
**RPE**	L Olfactory cortex	-17	9	-12	994	7.63	<.001	<.001
	R Precentral Gyrus	25	-24	54	3524	7.48	<.001	<.001
	L Superior Frontal Gyrus	-17	42	45	230	5.85	<.001	.010
	R Posterior-Medial Frontal	4	15	63	1979	-8.40	<.001	<.001
	R Inferior Frontal Gyrus (p. Triangularis)	55	18	15	1703	-7.27	<.001	<.001
	L Inferior Frontal Gyrus (p. Orbitalis)	-35	21	0	902	-6.77	<.001	<.001
	R Middle Temporal Gyrus	49	-36	0	258	-5.54	<.001	.005
	Cerebellum	-32	-54	-33	904	-5.44	<.001	<.001
**children > adults**	R Precuneus	10	-66	60	198	4.49	<.001	.021
	R Posterior-Medial Frontal	13	-3	57	333	4.43	<.001	.001
	R Middle Occipital Gyrus	31	-90	18	766	-5.30	<.001	<.001

*Note*. Only main peak per cluster reported. Regions were automatically labelled using the AnatomyToolbox atlas. x, y, and z = Montreal Neurological Institute (MNI) coordinates in the left-right, anterior-posterior, and inferior-superior dimensions, respectively. Thresholding: *t* > 2.672; *p* < .005; *df* = 52. L= left, R = right, RPE = reward prediction error.

##### 3.3.1.2 Prediction error

The RPE signal positively modulated brain activation in the bilateral striatum, a bilateral cluster including the pre- and post-central gyrus, and the left superior frontal gyrus for both groups combined. Brain activation in the bilateral posterior–medial frontal gyrus, the bilateral inferior frontal gyrus, the right middle temporal gyrus, and the bilateral middle cingulate gyrus was negatively modulated by the RPE signal for both groups combined. Children showed a stronger RPE signal in the bilateral precuneus and the right superior frontal gyrus. Adults showed a stronger signal in the right middle occipital gyrus (see Table 11 and Fig. 7B).

To identify activation patterns shared between adults and children, we conducted conjunction analyses examining common modulation of surprise and RPE during the match recognition task. These analyses revealed overlapping surprise-related activation in the left middle frontal gyrus, and RPE-related activation in the right precentral gyrus, bilateral anterior insula, and anterior cingulate cortex (ACC), consistent with the main effects reported in the whole-brain analyses (see Supplementary Material S3).

#### 3.3.2 Task-related effects in children (match recognition vs. discriminative choice)

##### 3.3.2.1 Surprise processing

**Fig. 8.**
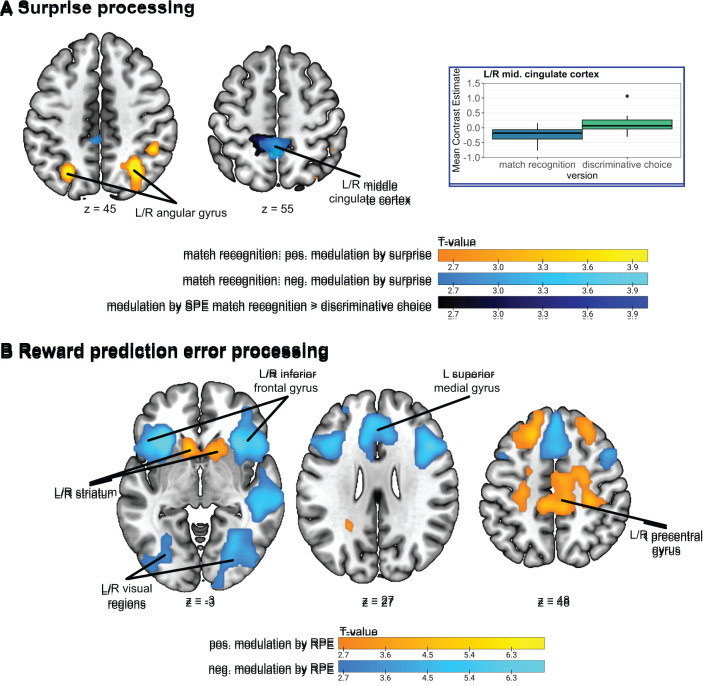
Task comparison: Comparison of brain activation during stimulus and feedback modulated by surprise and RPE between the two task versions. The upper panel (A) shows the positive and negative modulation of the BOLD signal by the surprise parameter in the match recognition task and the differences between the two task versions (in dark blue). The boxplot shows the mean extracted, whitened y values from SPM12 in the cluster where we found a significant difference between the two task versions. Match recognition task in blue, discriminative choice task in green. (B) The positive and negative modulation of the BOLD signal by the reward prediction error parameter for both tasks combined. Orange-yellow shows the positive modulation, light blue the negative modulation by the parameters. Cluster defining threshold *p* < .005 (unc.), *p*_FWEc_ = .05, family error-wise corrected. Pos. = positive, neg. = negative; mid. = middle, RPE = reward prediction error, L = left, R = right.

In the match recognition task, the BOLD signal in the bilateral angular gyrus was positively and in the bilateral middle cingulate cortex negatively modulated by the surprise signal. No modulation of the BOLD by surprise was found for the discriminative choice task. A two-sample t-test showed that the bilateral angular gyrus and the bilateral middle cingulate cortex were stronger modulated by the surprise signal in the match recognition compared with the discriminative choice task (see Fig. 8A and Table 12).

**Table 12.** Significant clusters modulated by surprise and RPE signals.

		MNI coordinates						
Contrast	Brain area	x	y	z	Cluster size	T-value	Peak level *p*_unc_	Cluster level *p*_FWE_
**surprise match recognition**	L Angular Gyrus	-26	-63	39	167	4.86	<.001	.046
	R Angular Gyrus	31	-63	45	378	4.55	<.001	<.001
	L Middle Cingulate Cortex	-11	-36	63	321	-4.03	<.001	.001
**surprise discriminative choice** **>** **match recognition**	L Middle Cingulate Cortex	-1	-36	51	432	4.17	<.001	<.001
**RPE**	R Olfactory cortex	19	6	-12	600	6.50	<.001	<.001
	L Paracentral Lobule	-14	-33	72	3770	5.99	<.001	<.001
	L Calcarine Gyrus	-5	-60	12	240	4.69	<.001	.010
	L Inferior Frontal Gyrus (p. Orbitalis)	-32	27	0	1430	-8.71	<.001	<.001
	R Inferior Frontal Gyrus (p. Orbitalis)	34	21	-9	1826	-7.35	<.001	<.001
	L Superior Medial Gyrus	-2	24	60	1728	-7.25	<.001	<.001
	R Fusiform Gyrus	28	-63	-6	977	-6.29	<.001	<.001
	R Middle Temporal Gyrus	52	-33	-3	405	-5.71	<.001	<.001
	L Cerebellum (Crus 1)	-38	-54	-30	737	-5.21	<.001	<.001

*Note*. Only main peak per cluster reported. Regions were automatically labelled using the AnatomyToolbox atlas. x, y, and z = Montreal Neurological Institute (MNI) coordinates in the left-right, anterior-posterior, and inferior-superior dimensions, respectively. Thresholding: *t* > 2.672; *p* < .005; *df* = 53. L = left, R = right, RPE = reward prediction error.

##### 3.3.2.2 Reward prediction error processing

In the match recognition task, the BOLD signal in the bilateral precentral gyrus and the right striatum was positively modulated by the RPE signal. The bilateral inferior frontal gyrus, bilateral posterior-medial frontal gyrus, and the bilateral fusiform gyrus were negatively modulated by the RPE signal. In the discriminative choice task, the BOLD signal was positively modulated by the RPE signal in the bilateral striatum, left middle frontal gyrus, and bilateral precuneus. The bilateral inferior frontal gyrus, right fusiform gyrus, and bilateral superior medial gyrus were negatively modulated by the RPE signal. A two-sample t-test showed no significant differences in RPE processing between the two task versions (see Fig. 8B and Table 12).

To assess neural commonalities across tasks, conjunction analyses were performed across both groups of children completing the match recognition and discriminative choice tasks. No shared modulation of surprise or positive RPE effects was observed; only negative RPE modulation emerged in the bilateral anterior insula and superior frontal gyrus. These results largely mirrored the main whole-brain findings (see Supplementary Material S3).

### 3.4 ROI analyses

We performed ROI analyses to further investigate the differences between the two groups and the two task versions in specific brain regions related to reward prediction error and surprise processing.

#### 3.4.1 Age-related effects in the match recognition task (adults vs. children)

We used the RPE and unexpected networks from Neurosynth to examine the effects of age group on RPE and surprise processing in specific ROIs. We did not observe a significant effect of group (adults or children) or modality (audio–visual or tactile–visual) in both networks.

#### 3.4.2 Task-related effects in children (match recognition vs. discriminative choice)

**Fig. 9.**
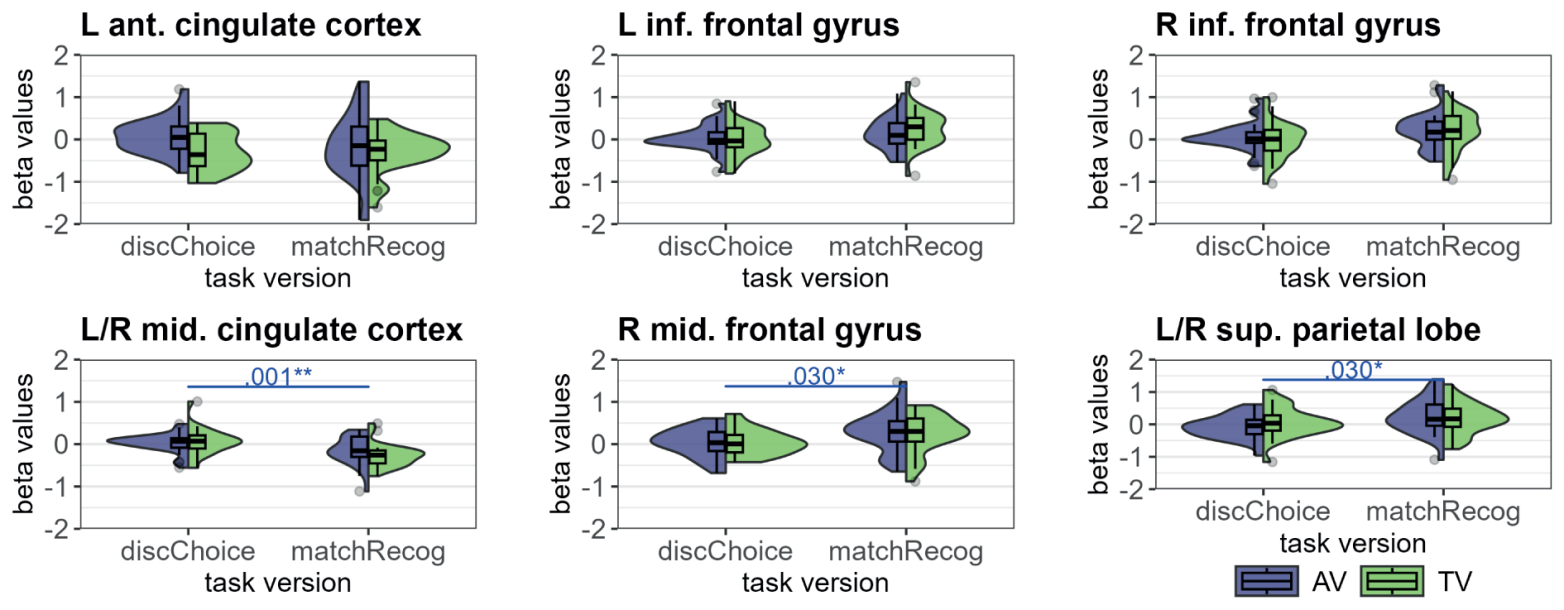
Beta values in the surprise cluster. *Note*. This figure shows the mean beta values for AV and TV feedback processing modulated by surprise in the surprise clusters from Section 3.3.1. AV (audio–visual) in blue, TV (tactile–visual) in green. *P* values are FDR corrected for multiple comparisons. discChoice = discriminative choice task, matchRecog = match recognition task, ant = anterior, inf = inferior, mid = middle, sup. = superior, R = right, L = left.

For the RPE, we used the network from neurosynth, and for surprise the clusters from the results described in Section 3.3.1. We did not observe any significant effects in the RPE network. In the surprise clusters, we observed a significant main effect of task version in the bilateral middle cingulate cortex, the right middle frontal gyrus, and the bilateral superior parietal lobe. In the middle cingulate cortex, the BOLD signal was negatively modulated by surprise in the match recognition task with no modulation in the discriminative choice task. In the middle frontal gyrus and the superior parietal lobe, the BOLD signal in the match recognition task was positively modulated by the surprise and no modulation in the discriminative choice task. The effect of task version showed a trend in the left and right inferior frontal gyrus with a positive modulation in the match recognition task and no modulation in the discriminative choice task. The effect of modality showed a trend in the left anterior cingulate cortex with a more negative modulation in TV runs than in AV runs (see Fig. 9 and Table 13).

**Table 13.** Main and interaction effects of task version (discriminative choice vs. match recognition) and modality (AV vs. TV) in the surprise clusters.

Model	Term	NumDF	DenDF	*F* Value	p_uncorr	*p*
L/R anterior	modality	1	109.00	6.06	0.015	.074.
cingulate gyrus	task	1	109.00	1.99	0.161	.483
	gender	1	109.00	0.42	0.520	.785
	modality × task	1	109.00	1.34	0.250	.522
L inferior	modality	1	55.00	0.23	0.631	.801
frontal gyrus	task	1	54.00	6.25	0.015	.074.
	gender	1	54.00	0.35	0.556	.785
	modality × task	1	55.00	1.29	0.261	.522
R inferior	modality	1	109.00	0.48	0.489	.785
frontal gyrus	task	1	109.00	5.35	0.023	.091.
	gender	1	109.00	0.02	0.898	.937
	modality × task	1	109.00	0.22	0.637	.801
L/R middle	modality	1	55.00	1.00	0.323	.596
cingulate gyrus	**task**	**1**	**54.00**	**19.11**	**0.000**	.001**
	gender	1	54.00	0.00	0.992	.992
	modality × task	1	55.00	1.72	0.195	.487
R middle	modality	1	109.00	0.06	0.807	.881
frontal gyrus	**task**	**1**	**109.00**	**9.15**	**0.003**	.030*
	gender	1	109.00	0.40	0.526	.785
	modality × task	1	109.00	0.14	0.711	.812
L/R superior	modality	1	109.00	0.19	0.667	.801
parietal lobe	**task**	**1**	**109.00**	**8.80**	**0.004**	.030*
	gender	1	109.00	2.70	0.103	.353
	modality × task	1	109.00	1.64	0.203	.487

*Note*. Type III Analysis of Variance Table with Satterthwaite’s method. Highlighted in bold are the significant effects after correction for multiple comparisons using the FDR method. Signif. codes: 0.001 “**” 0.01 “*” 0.05 “.” 0.1 “ ” 1. L = left, R = right, NumDF = numerator degrees of freedom, denDF = denominator degrees of freedom.

## 4 Discussion

This study aimed to examine and compare statistical and reinforcement learning between adults and children, in terms of both performance and neural processing. We also explored how statistical and reinforcement learning differ across two task versions with different response designs and difficulties. We employed computational modelling on behavioural data and integrated those with neuroimaging data to provide a comprehensive understanding of the cognitive and neural processing underlying multisensory learning between adults and children and task versions. Our results show that adults employ more complex learning strategies and an increased sensitivity to differences in values compared with children during multisensory associative learning. While neural representations of reward prediction errors are comparable between adults and children and the two task versions, neural surprise processing is still immature in children between 8.5 and 13 years old and dependent on task structure. The following section discusses the results and implications of our findings in detail, addresses the limitations of the current study, and suggests directions for future research in this area.

### 4.1 Greater value sensitivity in adults

We first investigated reaction times and accuracies as measures of performance. Both adults and children improved their performance within a run of the match recognition task, indicating learning, though adults showed stronger effects and overall higher accuracies, particularly in the audio–visual runs compared with tactile–visual runs. In children, reaction times decreased, and accuracies increased over time within a run for both task versions. Accuracies and reaction times did not differ between the discriminative choice task and the match recognition task.

We then applied computational models on the behavioural data to further investigate the learning involved in this task. Here, we expected lower learning rates in adults than in children due to the probabilistic nature of the task, because in this setting, higher learning rates would overestimate the effect of probabilistic trials and thus signify a less optimal updating strategy (Decker et al., 2015; Nussenbaum & Hartley, 2019). We could not confirm this expectation. This indicates that both groups adjust their beliefs based on the RPEs to a similar extent. However, we did observe shorter non-decision times and higher drift weights and drift rates for adults than for children. The longer non-decision times for children indicate slower encoding and response processes in children (Ratcliff & McKoon, 2008). The drift weight scales the captured differences between the presented beliefs (Pedersen et al., 2017) and, therefore, these results indicate higher sensitivity to value differences in adults than in children. This then also translates to the higher drift rates observed in adults and leads to faster information accumulation in adults. Previous studies showed increased inverse temperatures in the Softmax function in adults compared with children (Bolenz et al., 2017; Christakou et al., 2013; Decker et al., 2015; Nussenbaum & Hartley, 2019; Palminteri et al., 2016; Rodriguez Buritica et al., 2019), indicating more deterministic behaviour in adults and more probabilistic behaviour in children. The higher drift weights and rates observed here in adults, therefore, support several key findings: Adults demonstrate greater sensitivity to value differences and accumulate information more rapidly than children. Specifically, even when only small differences in evidence favouring one option were perceived, as indicated by the drift weight findings, adults tend to choose that option more frequently and confidently than children. This behaviour points to more deterministic decision making in adults.

In addition, model-fitting results revealed that adults more often employed advanced learning strategies during the MSL task. In adults, the transfer RW model was equally likely as the simple RW model to be the best fitting, whereas in children the simple RW model was more likely to be the best-fitting model. In the simple RW model, only the values of the presented and chosen options were updated and this information is not used to infer on the matching of other pairs. In contrast, in the transfer RW model, the information from the presented and chosen options is used to infer on other pairs. In the discriminative choice task, if the left symbol was correctly chosen, the value of this pair was increased and the value of the other pair decreased. In the match recognition task, if the participant learned that the presented pair was matching, its value was increased and the value for all other pairs including either one of the unimodal stimuli was decreased. This model represents a learning strategy in which participants not only update their expectations based on direct feedback but also transfer this information to infer and learn about other, not-presented options. Taken together, this implies that children overall seemed to employ a simpler learning strategy which may be less efficient compared with that employed by adults. This is supported by a stronger increase in accuracies over bins in adults compared with children. Additionally, we did not find significant differences between the two task versions regarding all model parameters in children. This suggests that the processing of stimuli, decision making and execution, and value updating seem to be comparable between task versions in children between 8 and 13 years old. These results indicate that learning occurred in both task versions, that the match recognition task is not inherently more difficult for the children in this age range, and that multisensory learning is comparable between task versions.

Next, we also investigated the effects of surprising trials on the reaction time. We found slower reaction times in more surprising trials for all groups and both task versions, as we expected based on the literature (Bedi et al., 2025; Kvålseth, 1987; Manning & Molesworth, 2023). These findings suggest that the processing of unexpected events and comparison with prior beliefs to reach a decision is slowed and potentially requires additional cognitive demands (Visalli et al., 2019).

In summary, we showed that adults exhibited faster information accumulation and greater sensitivity to value differences, aligning with previous research suggesting more deterministic behaviour in adults and more probabilistic behaviour in children. Additionally, multisensory processing and learning on a behavioural level do not differ in children between the two task versions. Finally, in both comparisons, surprising trials led to slower reaction times, reinforcing the role of expectation in decision processes.

### 4.2 Immature surprise processing in children

In our task, the brain activation in the inferior frontal gyrus and superior parietal lobule showed increased activation in surprising trials, whereas the anterior and middle cingulate cortex showed decreased activation in surprising trials during the match recognition task for adults and children combined. These findings are in line with the expectations based on previous studies and as further discussed below (Alexander & Brown, 2019; Hayden et al., 2011; Konovalov & Krajbich, 2016; O’Reilly, 2013; Visalli et al., 2019). These regions overlap nicely with the regions involved in the fronto-parietal network (Dosenbach et al., 2008) and the salience network (Dosenbach et al., 2008; Menon, 2015), which are both linked to cognitive control (Visalli et al., 2019). The fronto-parietal network, including the superior parietal lobule, precuneus, and inferior frontal gyrus, has been shown to apply to top–down control by trail-wise modification of task information (Visalli et al., 2019). Thus, it updates the beliefs about the world based on newly experienced observations. The anterior cingulate cortex together with the anterior insula forms the salience network involved in guiding behaviour based on identifying the relevant stimuli (Seeley et al., 2007). Group comparisons revealed a stronger response to the surprise signals in adults than in children in the precuneus which has been identified as a hub integrating information across various functional networks (Fornito et al., 2012; Leech et al., 2012). However, the activation in children was less broadly distributed across these two networks compared with adults since in this group only the angular gyrus and the middle cingulate were positively or negatively modulated by surprise, respectively. This suggests that surprise processing is still developing in middle childhood (Ren & Wang, 2023).

Of note, we did not observe any modulation of the BOLD response by the surprise signal in the discriminative choice task, neither in the whole-brain nor in the ROI analyses. In this task, surprise was manipulated by varying the occurrence of the non-matching symbol. The lack of modulation in the discriminative choice task for children may indicate that the modulation of surprise via the non-matching stimulus might not be sufficiently distracting to evoke a brain response. To further ensure that the absence of a surprise effect in the BOLD signal was not due to insufficient statistical power in the discriminative choice task, we repeated the analysis in a larger sample of 67 children (described in Raduner et al., 2025). This analysis (summarized in Appendix A3) likewise did not reveal significant neural surprise processing during the discriminative choice task.

These findings highlight a more widespread and efficient processing of surprise signals in adults in the salience and fronto-parietal network which is still immature during middle childhood. Overall, our results highlight the dynamic interplay between cognitive control and salience detection in processing unexpected events.

### 4.3 Uniform reward prediction error processing between adults and children and task versions

The brain activation modulated by the RPE corresponded to the findings from previous studies for both positive (Cohen & Ranganath, 2007; Fraga-González et al., 2025; McClure et al., 2003; O’Doherty et al., 2015; Pagnoni et al., 2002) and negative modulations (Fouragnan et al., 2018). We found no significant differences in brain regions typically associated with RPEs between adults and children completing the match recognition task nor between two task versions completed by age-, handedness-, and IQ-matched child groups. This was consistent across both whole-brain and ROI analyses and aligns with findings from previous studies (Hauser et al., 2015; Van Den Bos et al., 2012). Children, however, showed a stronger RPE response in the precuneus and the superior frontal gyrus as compared with adults. As stated earlier, the precuneus is seen as an integration hub between different functional networks (Fornito et al., 2012; Leech et al., 2012). One study showed that the connectivity between the precuneus and the FPN increased during task performance across three different decision-making tasks (incentive response-time task, financial decision-making task, risk valuation task) compared with rest (Utevsky et al., 2014). This indicates that the multisensory learning task might be more demanding for children than for adults, which is supported by the weaker increase in accuracies over bins and simpler learning strategies in children than in adults. In turn, this then leads to a broader processing of RPEs in children’s brains in the precuneus and FPN. These findings suggest that while the core neural mechanisms of RPE processing are similar across ages, children may recruit additional brain regions, possibly reflecting increased cognitive demands during multisensory learning.

In summary, the brain activation associated with RPE processing here is consistent with the literature and does not yield any significant differences in key brain regions (e.g., Corlett et al., 2022) between adults and children or task versions. This suggests that the fundamental neural mechanisms underlying RPE processing may be shared between adults and children. However, children may engage additional brain regions (such as the superior frontal gyrus and precuneus) to support the increased cognitive demands associated with multisensory learning. Taken together with the findings from surprise processing, our results show that both learning signals are present in parallel during the performance of a simple associative learning task, in both adults and children.

### 4.4 Implications and limitations

Our findings suggest two key implications for our understanding of statistical and reinforcement multisensory learning during typical development and for future research. First, they demonstrate that neural processes related to reward prediction errors are similarly engaged between adults and children and task versions (Corlett et al., 2022; Hauser et al., 2015; Van Den Bos et al., 2012), with only minor age-related differences. These differences likely reflect the increased cognitive demands posed on children during these multisensory learning tasks. This opens the door for future research to investigate reward prediction errors across different groups (e.g., age groups or also patient groups) by utilizing tasks that are adjusted to the individual skill levels within or across groups.

Second, our study showcases how the approach of studying reinforcement and statistical learning in one integrated design (Bedi et al., 2025) can provide a simple yet informative alternative to more complex, multi-stage paradigms (e.g., Möhring & Gläscher, 2023) or to using two separate tasks for each form of learning. This approach allows for a more comprehensive understanding of how both types of learning interact in children, who are still refining their statistical learning abilities. However, our results highlight that the specific task design may play a crucial role in the expression of the statistical learning processes, which underscores the importance of task calibration when examining developmental differences or designing interventions to enhance learning.

Third, while the findings of the present study provide insights into multisensory learning mechanisms across different task variants, our study employed a between-subjects design. This choice was driven by practical constraints in scanning children, and although it allowed us to maintain high data quality within each task version, a within-subject comparison may offer a more direct assessment of behavioural and neural differences between task variants and could be considered in future research.

Last, since surprise processing seems to be still immature in children compared with that in adults, an interesting topic for future longitudinal studies would be to investigate developmental changes within the processing of statistical regularities of the environment.

Although gender was included as a covariate in the analyses to account for potential effects, the gender imbalance in the adult sample may still limit the generalizability of findings. This should be considered when interpreting group-level comparisons.

### 4.5 Conclusion

In summary, our findings provide valuable insights into the interplay between age, task complexity, and learning in multisensory environments. The results of our computational modelling revealed important age-related differences in decision-making dynamics, such as faster information accumulation and increased sensitivity to value differences in adults. Additionally, while reward prediction error processing appeared consistent between adults and children, children exhibited greater engagement of brain regions involved in cognitive control and integration, suggesting increased cognitive demands during multisensory learning. Further, we observed still immature surprise processing in children, with this signal depending on the specific task that was used. These findings underscore that parallel studies of different types of multisensory learning offer interesting insights into development of multisensory processing, but they also highlight the importance of task design for such studies and suggest that future research should focus on tailoring tasks to better capture these processes.

## Supplementary Material

Supplementary Material

## Figures and Tables

**Appendix Fig. A1.**
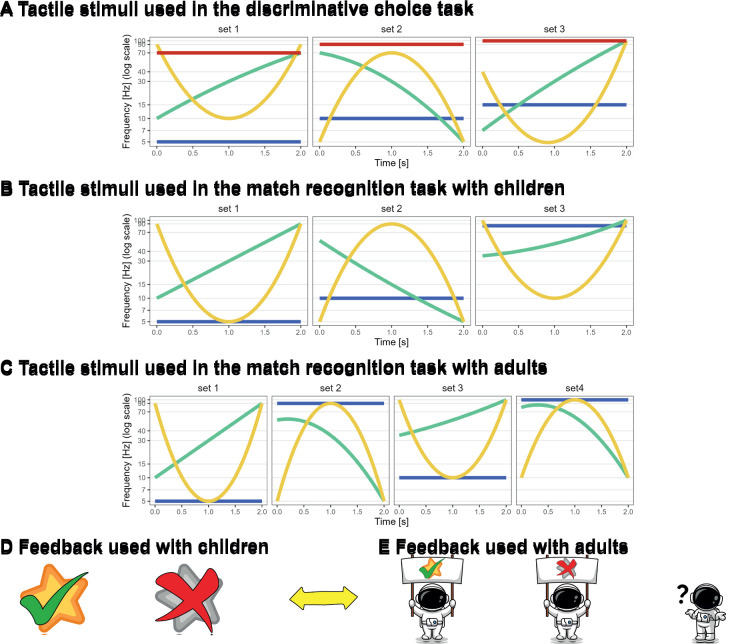
Depiction of the stimuli used in this study. (A) The frequency patterns for the three tactile stimulation sets used in the discriminative choice task in children, (B) in the match recognition task in adults, and (C) in children. (D) The feedback used for the tasks used with children and (E) for the task used with adults.

## Data Availability

The data (no raw or preprocessed neuroimaging data) and code for this publication are available on https://doi.org/10.17605/OSF.IO/DESZ2.

## Appendix A1. Stimuli

All visual and auditory stimuli used in this study are uploaded here: https://doi.org/10.17605/OSF.IO/DESZ2. Appendix Figure A1A, B, and C depict the tactile vibration patterns and Appendix A1D and E the stimuli used as feedback.

## Appendix A2. Trial Structures and Probabilistic Feedback

We developed various trial structures for the discriminative choice and match recognition task in children in order to adjust the task difficulty to the individual performance. In the easy trial structure, each auditory or tactile stimulus may be repeated once in two consecutive trials and each third (for the match recognition task) or fourth (for the discriminative choice task) could have one repetition (e.g., auditory stimulus A appeared in trials 7 and 8). But all of the auditory and tactile stimuli were presented equally often during a run, despite the subsequent repetitions. There was no consecutive repetition of tactile or auditory stimuli in the hard trial structure. For the adults, only hard trial structures were used. Additionally, we ensured that the exposure to the multisensory stimulus combinations was balanced across each run by balancing the presentation of the auditory and tactile stimuli across one-third (for the match recognition task) or fourth (for the discriminative choice task) of each run.

Furthermore, we use probabilistic feedback in our tasks in order to adjust the task difficulty to the individual performance in which the participants received incorrect feedback, such as negative feedback following a correct response or vice versa. We predetermined in which trials incorrect feedback was given so that the incorrect feedback was spread across all matching multisensory combinations. This ensured that the difficulty of each multisensory combination was balanced. We included three levels of difficulty: 5% incorrect feedback (2 trials per run), 10% incorrect feedback (4 trials per run), or 20% incorrect feedback (8 trials per run). For the children, the trials with incorrect feedback were also balanced across task thirds (for the match recognition task) or fourths (for the discriminative choice task). For the match recognition task, the two trials were in the second and last third for 5%, one in the first and second and two in the last third for 10%, two in the first third and three in each the second and last third for 20% incorrect feedback. For the discriminative choice task, the trials were in the second and last fourth for 5%, one in each fourth for 10%, and two in each fourth for 20% probabilistic feedback. For the adults, 30% probabilistic feedback was also possible (13 trials per run). Here, trials with incorrect feedback were randomly attributed at the beginning of each run but balanced across 10 consecutive trials (e.g., with 30% probabilistic feedback, 3 trials were randomly selected from the first 10 trials, then 3 from the second 10 trials, and so on). While all three levels of probabilistic feedback could be paired with the hard trial structure, the easy trial structure was always paired with 5% probabilistic feedback. In total, we had four hard trial structure for adults, and eight easy and eight hard for the children, all of which were balanced within and between subjects. All behavioural runs were completed with the hard trial structure and no probabilistic feedback for children, and 20% probabilistic feedback for adults. In the MR session, the easy trial structure with 5% probabilistic feedback was used if the pre-run task accuracy was <60%. The hard trial structure with 5% probabilistic feedback was applied if the pre-run accuracy was between 60% and 80%. Finally, the hard trial structure with 10% probabilistic feedback was employed if the pre-run performance was >80%.

## Appendix A3. Surprise Analyses in a Larger Sample

To verify the absent neural correlates of the surprise signal, this analysis was repeated in the sample of 67 children (*M* = 8.35 ± 1.82 years, 36 girls) completing the discriminative choice task from Raduner et al. (2025). The Shannon surprise was calculated for each trial and then added as a parametric modulator on the stimulus onset regressor. We did not observe any significant clusters showing a positive or negative modulation by the surprise values (*p*_CDT_ < .005).
