# Supplementary material for: Neural and behavioural differences in multisensory statistical and reinforcement learning across development and task variants
Source: Imaging Neurosci (Camb). 2026 Feb 2;4:IMAG.a.1117. doi: 10.1162/IMAG.a.1117 (PMC12865814; doi:10.1162/IMAG.a.1117)
Supplement: Supplementary Material [file IMAG.a.1117_supp.pdf]

Supplementary Material

S1 Baseline Cognitive Characteristics of Child Groups

To ensure comparability between the two child groups, we assessed a range of baseline cognitive abilities, including reading fluency, multisensory detection, working memory, processing speed, rapid automatised naming, sustained attention, inhibitory control, and divided attention. No significant differences were observed between the groups on any of these measures. Detailed results are presented in Table S1.

Table S1

Baseline cognitive characteristics of children groups

	match recognition	discriminative choice	*t* value	*p*	95% CI
Word reading fluency [PR]	53.86	44.57	1.20	.234	[-6.17, 24.75]
Non-word reading fluency [PR]	51.07	40.52	1.39	.172	[-4.72, 25.83]
Relative multisensory gain [%]	4.90	0.15	1.58	.120	[-1.28, 10.78]
Corsi block tapping [raw]	18.11	16.69	1.83	.073	[-0.14, 2.97]
Digit span [raw]	16.04	15.59	0.54	.594	[-1.23, 2.13]
Rapid automatised naming [T]	50.00	49.17	0.36	.723	[-3.84, 5.49]
Processing speed [T]	48.79	49.86	-0.47	.640	[-5.67, 3.51]
Sustained attention RT [PR]	55.46	48.62	0.87	.390	[-8.97, 22.66]
Go/No-Go RT [PR]	51.39	49.59	0.23	.822	[-14.23, 17.84]
Divided attention aud. RT [PR]	25.46	30.89	-0.77	.446	[-19.59, 8.74]
Divided attention vis. RT [PR]	40.57	49.21	-1.21	.231	[-22.94, 5.65]

Note. The two child groups were compared in their baseline cognitive function. Word and non-word reading fluency was measured using the “Salzburger Lese- und Rechtschreibtest” (SLRT-II, Moll & Landerl (2010)). Relative multisensory gain scores were measured in a multisensory simple detection task and indicate the gain on the rection times in percent during multisensory compared to unisensory stimulus presentation (Denervaud et al., 2020). Visual and verbal working memory were assessed using the corsi block tapping (Corsi, 1972; Farrell Pagulayan et al., 2006) and digit span (Wechsler, 2017), respectively. Here, the numbers indicate the raw sum scores for forward and backward span. Rapid automatised naming and processing sp(Grob & Hagmann-von Arx, 2018)ts from the IDS-2 (Grob & Hagmann-von Arx, 2018). Sustained attention, Go/No-Go performance and divided attention were measured using the corresponding subtests from the KITAP (Zimmermann et al., 2002). PR = percentile, T = T value, aud. = auditory, vis. = visual.

S2 Modelling: simulation and recovery

Before fitting the different variations of the RW model, we simulated data and recovered parameters for each of the model variation and task designs, as recommended by Wilson & Collins (2019). To do so, we created 100 data sets for each

model variation and each task design where one of the possible trial structures was selected randomly. Next, for each free parameter values were randomly chosen from a uniform distribution $U \sim uniform(0,1)$. The values for each learning rate ranged from 0 to 1, for non-decision time τ from 0.3 to 3.0, for the drift weight v_{mod} from 0 to 15, and the decision boundary a from 1 to 5. These random parameters were then used to simulate artificial responses and reaction times to create artificial, simulated data sets, which were then used to estimate the parameters. Finally, the recovered parameters were compared to the simulated values for each data set and the results are summarised in **Figure S1.1-S1.4**. Results show successful high and significant correlations between all free parameters except the η_{+}^{O} in the match recognition task (see **Figure S1D**).

Figure S2.1

Comparison of simulated vs. fitted parameters for the simple Rescorla-Wagner model

A Scatter plot for simulated and recovered parameter values

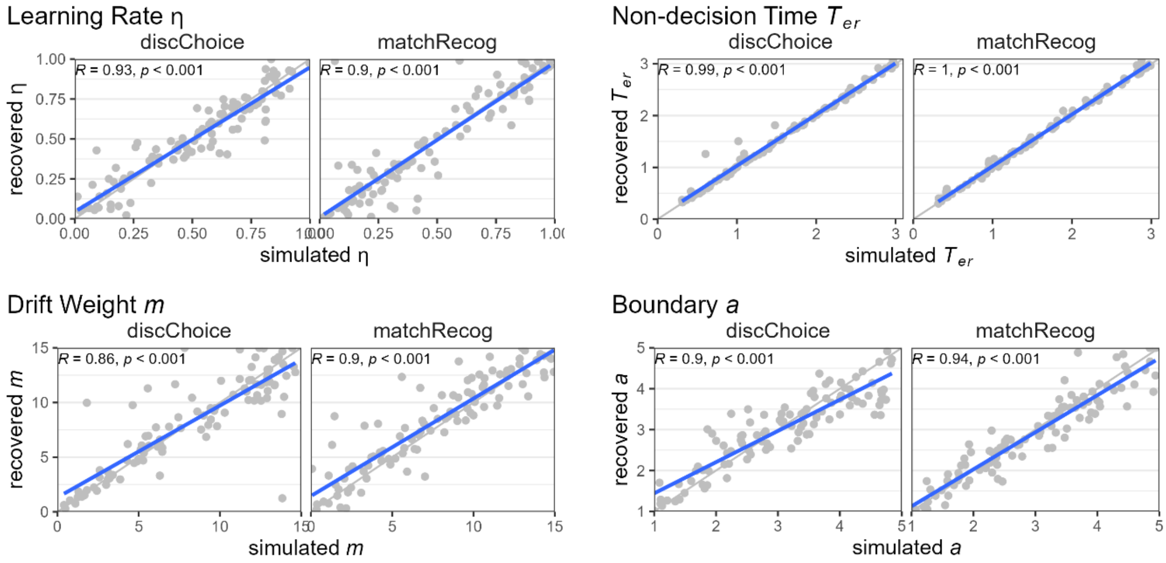

B Correlations between all simulated and recovered parameters

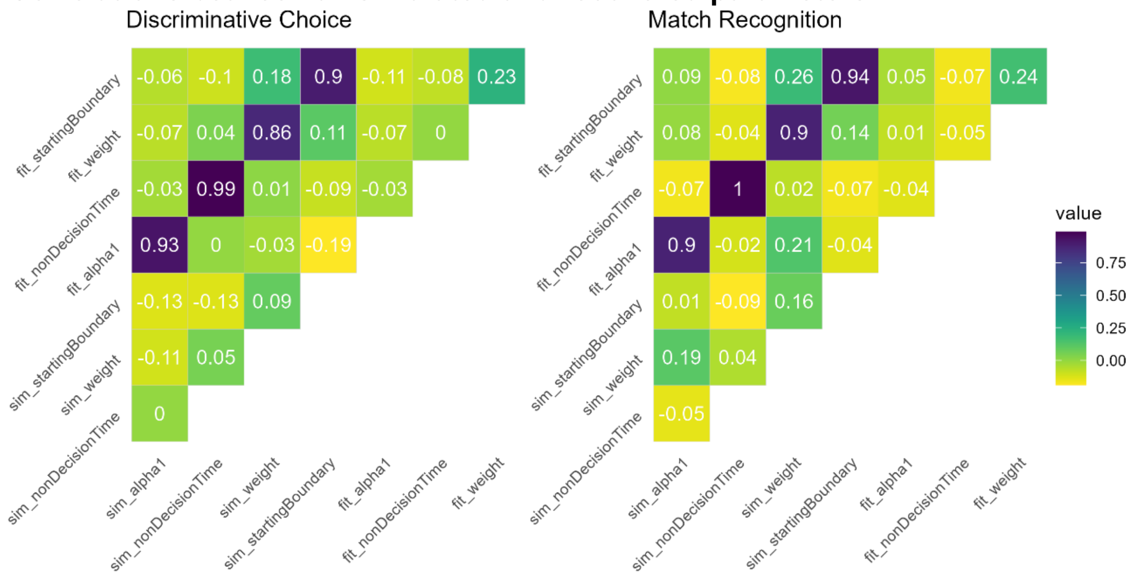

Note. The correlations between simulated and fitted parameters for the simple Rescorla-Wagner model are depicted here. In panel **A**, the correlations between simulated and recovered parameters are shown. Panel **B** shows the correlations between all the simulated and recovered parameters. discChoice = discriminative choice task; matchRecog = match recognition task.

Figure S2.2

Comparison of simulated vs. fitted parameters for the simple asymmetric Rescorla-Wagner model

A Scatter plot for simulated and recovered parameter values

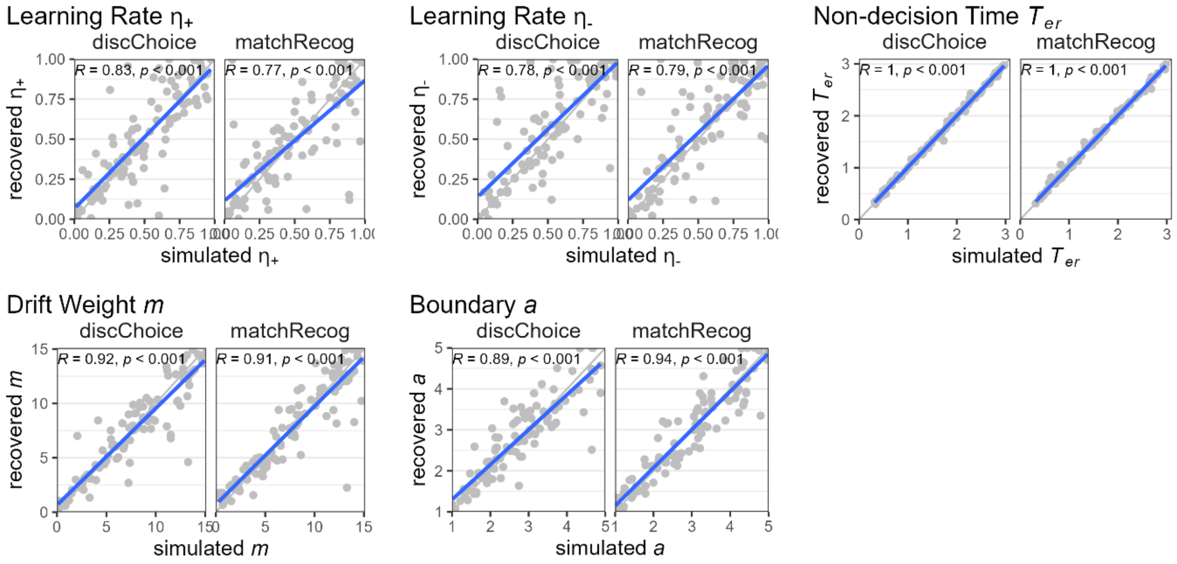

B Correlations between all simulated and recovered parameters

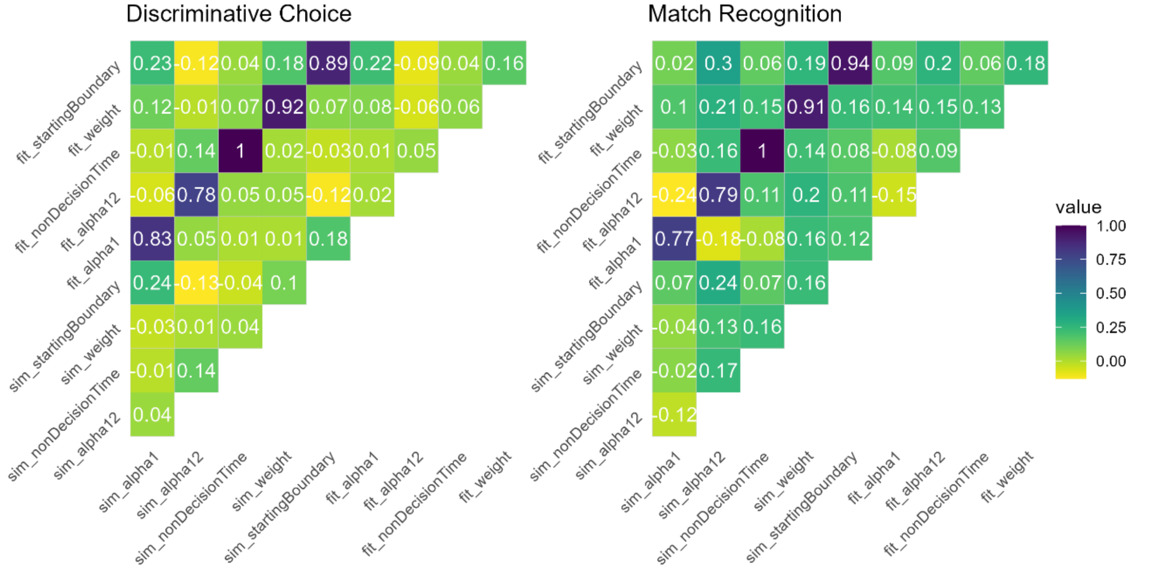

Note. The correlations between simulated and fitted parameters for the simple asymmetric Rescorla-Wagner model are depicted here. In panel **A**, the correlations between simulated and recovered parameters are shown. Panel **B** shows the correlations between all the simulated and recovered parameters. discChoice = discriminative choice task; matchRecog = match recognition task.

Figure S2.3

Comparison of simulated vs. fitted parameters for the transfer Rescorla-Wagner model

A Scatter plot for simulated and recovered parameter values

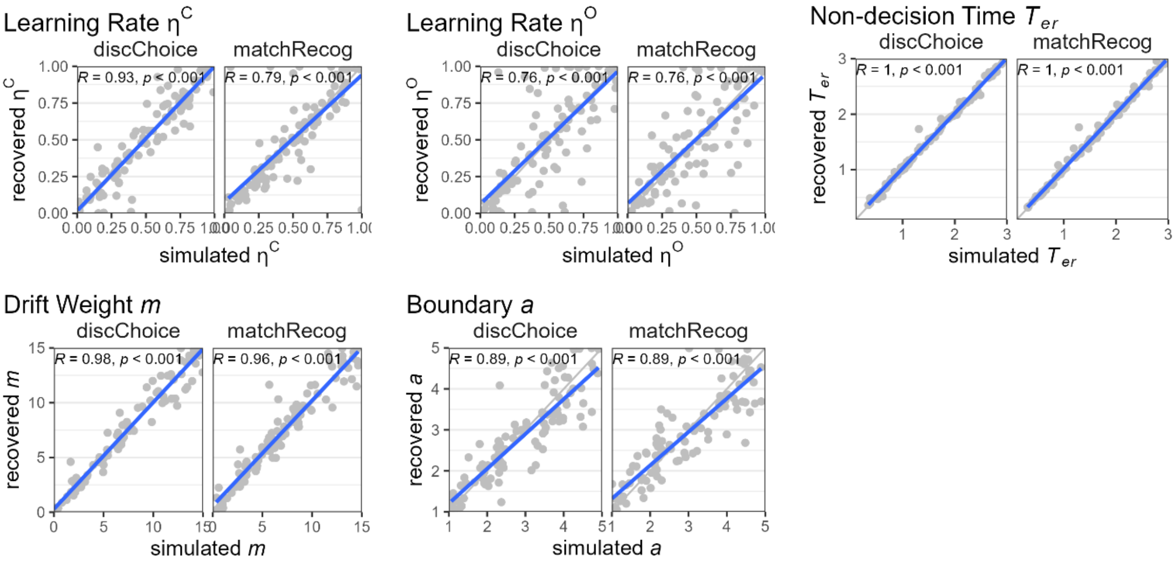

B Correlations between all simulated and recovered parameters

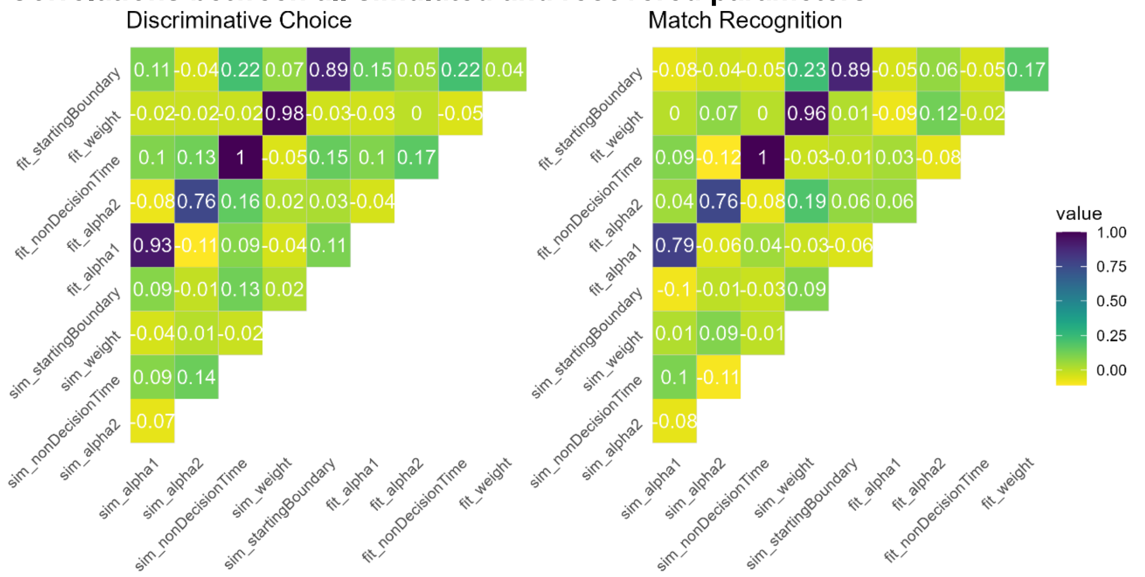

Note. The correlations between simulated and fitted parameters for the transfer Rescorla-Wagner model are depicted here. In panel **A**, the correlations between simulated and recovered parameters are shown. Panel **B** shows the correlations between all the simulated and recovered parameters. discChoice = discriminative choice task; matchRecog = match recognition task.

Figure S2.4

Comparison of simulated vs. fitted parameters for the transfer asymmetric Rescorla-Wagner model

A Scatter plot for simulated and recovered parameter values

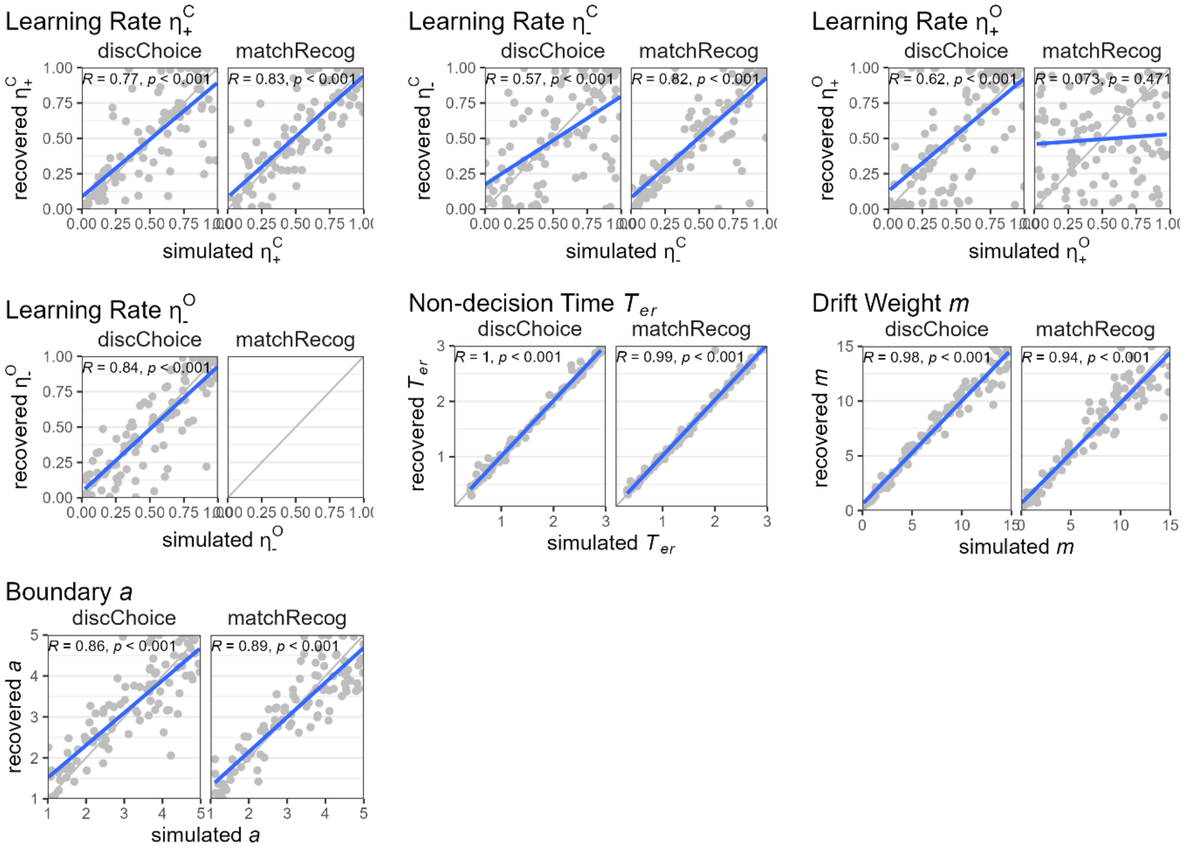

B Correlations between all simulated and recovered parameters

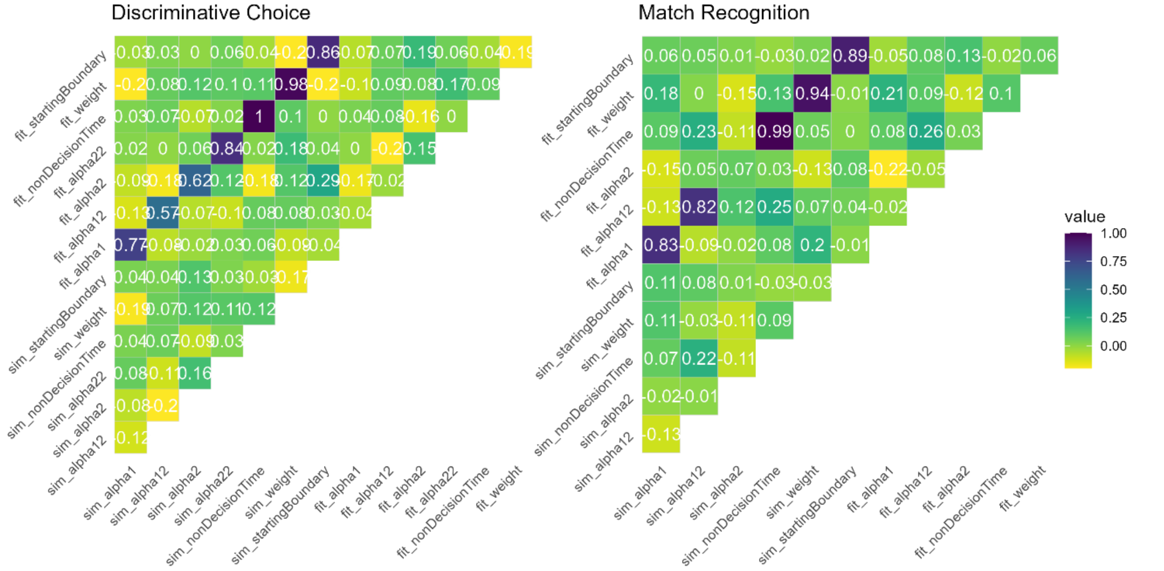

Note. The correlations between simulated and fitted parameters for the transfer asymmetric Rescorla-Wagner model are depicted here. In panel **A**, the correlations between simulated and recovered parameters are shown. Panel **B** shows the correlations between all the simulated and recovered parameters. discChoice = discriminative choice task; matchRecog = match recognition task.

S3 Conjunction Analyses

To further investigate task and age effects, conjunction analyses were conducted to examine shared neural activation associated with positive and negative modulation of surprise and reward prediction errors (RPE). Analyses were performed (a) across adults and children completing the match recognition task, and (b) across both groups of children completing the discriminative choice and the match recognition task.

Results revealed convergent activation patterns across age groups and task versions, indicating shared neural correlates of surprise and RPE processing. Specifically, surprise modulated activation in the left middle frontal gyrus in both adults and children during the match recognition task. RPE showed positive modulation in the right precentral gyrus and negative modulation in the bilateral anterior insula and the anterior cingulate cortex. Across both groups of children, no shared modulation of surprise or positive modulation of RPE was observed; only negative RPE modulation emerged in the bilateral anterior insula and the superior frontal gyrus.

Overall, these findings are consistent with the main whole-brain results and support the robustness of the identified activation patterns across groups and task versions.

Table S3

Significant clusters modulated by surprise and RPE signals

Contrast	Brain Area	MNI Coordinates x	y	z	Cluster size	T-value	Peak-level p_{unc}	Cluster-level p_{FWE}
**Match Recognition Adults ∩ Match Recognition Children**								
**surprise**	L Middle Frontal Gyrus	-41	3	33	168	4.02	<.001	.040
**RPE**	R Precentral Gyrus	25	-24	57	920	4.77	<.001	<.001
	R Inferior Frontal Gyrus	55	18	15	609	-5.13	<.001	<.001
	R Anterior Cingulum Cortex	1	15	66	575	-5.02	<.001	<.001
	L Anterior Insula	-32	21	0	239	-4.51	<.001	.008
**Discriminative Choice in Children ∩ Match Recognition in Children**								
**-RPE**	L Inferior Frontal Gyrus	-32	30	0	462	-5.90	<.001	<.001
	R Superior Frontal Gyrus	1	33	54	637	-4.83	<.001	<.001
	R Anterior Insula	52	21	-6	578	-4.38	<.001	<.001

Note. Only main peak per cluster reported. Regions were automatically labelled using the AnatomyToolbox atlas. x, y, and z = Montreal Neurological Institute (MNI) coordinates in the left-right, anterior-posterior, and inferior-superior dimensions, respectively. Thresholding: $t > 2.672$; $p < .005$; df = 52. L= left, R = right, RPE = reward prediction error.

References

Corsi, P. M. (1972). *Human memory and the medial temporal region of the brain.*

Denervaud, S., Gentaz, E., Matusz, P. J., & Murray, M. M. (2020). Multisensory Gains in Simple Detection Predict Global Cognition in Schoolchildren. *Scientific Reports*, *10*(1), 1394. https://doi.org/10.1038/s41598-020-58329-4

Farrell Pagulayan, K., Busch, R., Medina, K., Bartok, J., & Krikorian, R. (2006). Developmental normative data for the Corsi Block-Tapping task. *Journal of Clinical and Experimental Neuropsychology*, *28*(6). https://doi.org/10.1080/13803390500350977

Grob, A., & Hagmann-von Arx, P. (2018). *IDS-2-Intelligence and Development Scales-2. Intelligenz-und Entwicklungsskalen für Kinder und Jugendliche-Testkoffer*. Hogrefe.

Moll, K., & Landerl, K. (2010). *Lese-und Rechtschreibtest: SLRT-II: Weiterentwicklung des Salzburger Lese-und Rechtschreibtest (SLRT)*. Huber.

Wechsler, D. (2017). Wechsler Intelligence Scale for Children–Fifth Edition (WISC-V)(dt. Bearbeitung durch F. Petermann). In *Frankfurt a. M.: Pearson Assessment*.

Wilson, R. C., & Collins, A. G. E. (2019). Ten simple rules for the computational modeling of behavioral data. *ELife*, *8*. https://doi.org/10.7554/eLife.49547

Zimmermann, P., Fimm, B., & Gondan, M. (2002). *Testbatterie zur aufmerksamkeitsprüfung für kinder:(KiTAP);[das schloß der geister]*. Psytest.
